# Projected heat stress challenges and abatement opportunities for U.S. milk production

**DOI:** 10.1371/journal.pone.0214665

**Published:** 2019-03-28

**Authors:** Kpoti M. Gunn, Michael A. Holly, Tamie L. Veith, Anthony R. Buda, Rishi Prasad, C. Alan Rotz, Kathy J. Soder, Anne M. K. Stoner

**Affiliations:** 1 United States Department of Agriculture–Agricultural Research Service, Pasture Systems and Watershed Management Research Unit, University Park, Pennsylvania, United States of America; 2 Crop, Soil, and Environmental Sciences, Auburn University, Auburn, Alabama, United States of America; 3 Climate Science Center, Texas Tech University, Lubbock, Texas, United States of America; University of Illinois, UNITED STATES

## Abstract

Cost-effective heat mitigation strategies are imperative for maintaining milk production and dairy farm profitability in the U.S. with projected climate change. This study investigated the cost-effectiveness of four heat abatement strategies, including Minimal (open barn or shading), Moderate (forced ventilation), High (fans and misting), and Intense (air conditioning). Heat stress and subsequent impacts on milk production per cow were predicted across nine climatic regions in the U.S. for early (2015 to 2034), mid (2045 to 2064) and late (2081 to 2100) 21^st^ century, using downscaled climate projections. Heat abatements were used to adjust predicted milk production losses and illustrate the potential to reduce milk production losses due to heat stress. Economic analysis included a cost-benefit ratio calculation associated with the implementation of each heat abatement. Results showed that milk production losses were expected to accelerate across the U.S. at a mean rate of 174±7 kg/cow/decade, with the fastest rate in the Southeast region. Relative to Minimal heat abatement, Moderate, High, and Intense heat abatements increased annual milk production per cow by 3%, 4%, and 6% during early-21^st^ century, 3%, 6%, and 11% during mid-21^st^ century, and 3%, 8%, and 21% during late-21^st^ century, respectively. The cost effectiveness of different heat abatement strategies generally increased with subsequently stronger heat abatements. In mid- and late-21^st^ century, mean annual net values of High and Intense heat stress abatement implementation approached -$30 to $190 /cow and -$20 to $590 /cow, respectively, with the largest net annual benefit in late-21^st^ century under Intense abatement. Findings from the study demonstrate the value of using downscaled climate projections to shed light on local and regional strategies to abate heat stress on cattle and mitigate potential milk production losses due to climate change.

## Introduction

Projected increases in temperature and humidity across the U.S. [1–4] amplify the dairy industry’s need for proactive heat abatement. The U.S. dairy industry is principally comprised of large confinement dairy farms (> 400 cows), which contribute two thirds of the U.S. milk supply [5] and up to one third of the world’s milk production [6]. Milk products in the U.S. yielded $35.5 billion in revenue in 2012 (9% of total U.S. agricultural products sold) [7]. As ambient temperatures and relative humidity levels rise above a cow’s thermoneutral zone, energy and nutrients otherwise available for growth, reproduction, and milk production must be diverted to regulate body temperature, thus resulting in milk production losses [8,9]. Overall, heat stress costs the U.S. dairy industry approximately $897 to $1,500 million/year in revenue [10].

Previous climate change studies highlighting the role of heat stress on dairy production have focused on direct impacts (climate change vs. heat stress, climate change vs. animal health, climate change vs. animal growth and / or production, etc.) rather than the mitigating potential of heat abatement strategies. Initial studies explored the impacts of climate change on the temperature humidity index (THI) [11,12]; further studies explored the impacts of heat stress on milk production [10,13–21]. Results from these studies suggest that the additional heat stress caused by global warming could increase milk production losses for the average U.S. dairy farm from 0.6% in 2010 to 1.4% in 2030 and may reach more than 2% in some states [16]. Mauger et al. [15] found that milk production per cow would likely decrease as the number of heat stress days increased over the 21^st^ century; however, only humid subtropical regions were expected to be greatly impacted.

Several studies in the past 40 years have discussed methods to abate dairy heat stress under current or future climate conditions [10,22–29]. In particular, St-Pierre et al. [10] introduced the technical and economic implications of different housing-related abatement methods. They found that some form of abatement in addition to the simple use of mechanical ventilation is necessary for the U.S. dairy industry to prevent economic losses. However, their analysis was based on past climate data, starting from the beginning of the 20^th^ century, and did not account for the climate variability expected in the 21^st^ century. Additionally, St-Pierre et al. [10] explored the opportunity cost of heat abatement at the state level and did not analyze the economic feasibility of different levels of heat abatement.

Currently, there is limited economic information to support dairy producers in decision making regarding heat abatement in a changing climate. A comprehensive cost-benefit analysis of dairy heat abatement strategies in the U.S. over this century will help dairy producers know the cost-efficacy of heat abatement to sustain production. Accordingly, our objectives were to examine potential heat stress variation and to evaluate the effectiveness of housing-based heat abatements to reduce milk production losses under a changing climate. This analysis was conducted for selected locations within nine climatic regions in the U.S. Understanding the local and regional effectiveness of different heat abatements enabled an assessment of their economic feasibility and determined the most effective strategies for the long term. Findings from this analysis will contribute to ongoing efforts by USDA-ARS’s Dairy Agroecosystems Working Group (DAWG), which seeks to address the national grand challenge of advancing our knowledge of U.S. dairy adaptation strategies in a rapidly changing climate [30].

## Materials and methods

**Fig 1:**
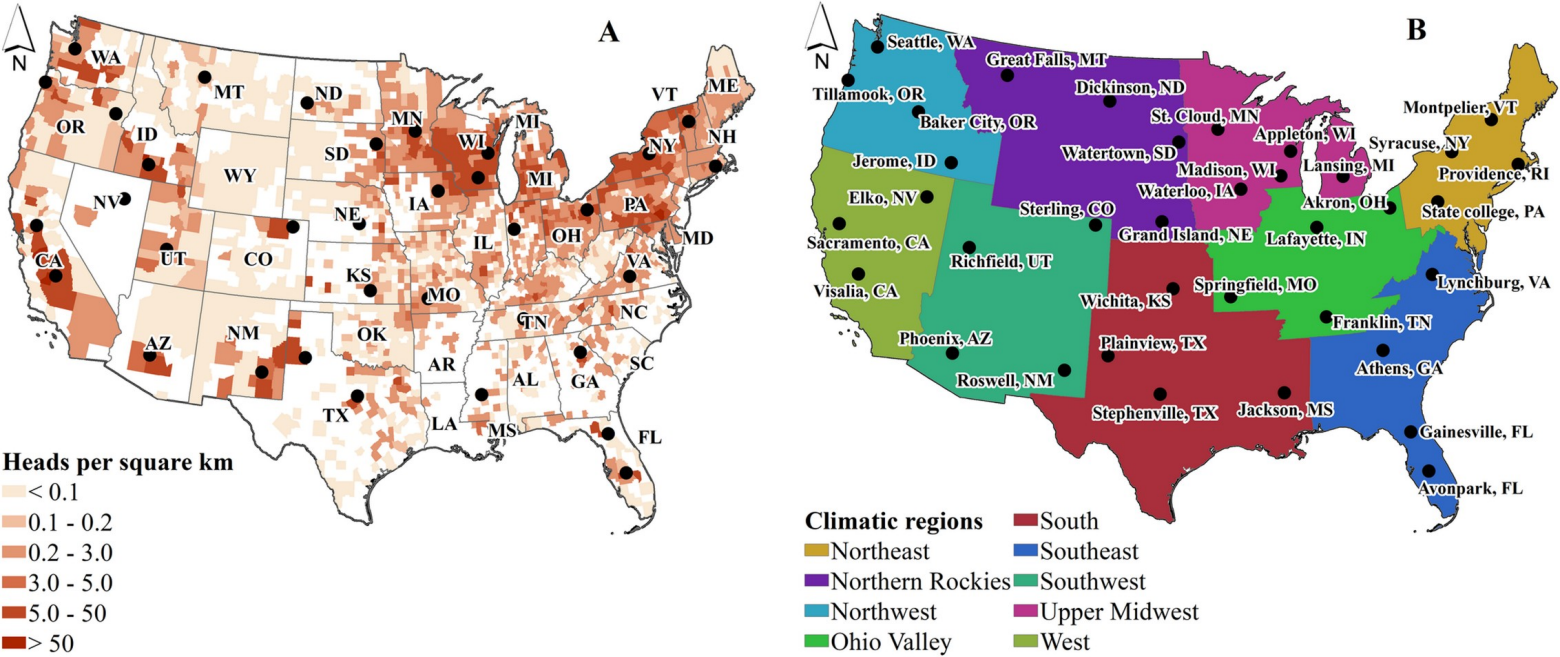
2012 U.S. dairy cow spatial density (A) and climatic regions (B) with study locations (Cow headcounts and boundaries data: [31–33]).

We selected 36 locations (Fig 1A) representing concentrated dairy production areas across nine U.S. regions (3 to 4 sites/region) that are climatically consistent and useful for identifying trends and geospatial distributions in climate [31] (Fig 1B). We selected three periods in the 21^st^ century [early (2015 to 2034), mid (2045 to 2064), and late (2081 to 2100)] and included one period in the 20^th^ century (1981 to 2000) to serve as a benchmark for projected changes. Future milk production losses per cow from heat stress were estimated using site-specific climate projections and four levels of housing heat abatement. An economic analysis of housing-based heat abatements was completed using cost-benefit ratios.

### Climate input

Representative Concentration Pathways (RCPs) are standardized scenarios of greenhouse gas concentrations used worldwide to explore the potential future impacts of climate change [34]. RCP 8.5 represents the most extreme scenario under which a business-as-usual emission pathway is pursued and resultant concentrations of atmospheric greenhouse gases reach nearly 1370 ppm CO_2_-eq by the end of the 21^st^ century [34]. Up to 2008, observations have indicated that greenhouse gas emission trends have not diverted much from the highest emission scenario [35]. Therefore, we selected RCP 8.5 as the most likely future greenhouse gas emission scenario and used output from nine global climate models (GCMs) (S1 Table) from the Coupled Model Intercomparison Project 5 (CMIP5) archive [36], representing the full range in climate sensitivity, which is the models’ climate response to a doubling in atmospheric CO_2_. Daily maximum, minimum, and mean gridded projections from the nine GCMs were statistically downscaled using the Asynchronous Regional Regression Model [37], which translates gridded GCM projections, with a typical resolution on the order of one hundred kilometers or more, into point-scale climate data directly relevant to and reflective of historical conditions at each location.

We used the method developed by Key and Sneering [16] to project daily dew point temperatures for the 21^st^ century, assuming that daily dew point temperature can be approximated by daily minimum temperature and that the contemporary difference between monthly minimum temperature and monthly dew point temperature is conserved in the future. Using the projected daily mean temperature and dew point temperature, we projected daily mean relative humidity using the Clausius-Clapeyron approximation for saturation vapor pressure and the assumption of a fixed enthalpy of vaporization [38]. Details of daily mean dew point temperature and relative humidity projections for the 21^st^ century are provided in S1 File. To evaluate the accuracy of the daily dew point temperature predictions, we compared the predictions to values reported by PRISM for the historical periods 2000 to 2010 [39], and found that predicted monthly and daily dew point temperature matched well with the observations (S1 Fig).

### Temperature humidity index (THI)

The degree of comfort for mammals like dairy cows has traditionally been evaluated using THI, which is estimated from dry bulb and wet bulb temperatures, dew point temperatures, or relative humidity [20]. In this study, we calculated THI using the method proposed by Yousef [40] for cattle [Eqs (1) and (2)] to accommodate the available projected data. This THI method uses daily mean dew point temperature (Tdew_d_) as a proxy for daily relative humidity, and was applied by Key and Sneering [16] to evaluate climate change impacts on dairy production across the continental U.S. We used maximum and minimum daily temperature (Tmax_d_ and Tmin_d_) to estimate maximum and minimum daily THI (THImax_d_ and THImin_d_):

$$THImax_{d}=Tmax_{d}+0.36\;Tdew_{d}+41.2$$ (1)

$$THImin_{d}=Tmin_{d}+0.36\;Tdew_{d}+41.2$$ (2)

### Housing-based heat abatement modeling

Several heat abatement strategies, including physical modification of animal environment, diet manipulation, genetic selection and hormonal treatment [28,41] are known to alleviate heat-related dairy production losses. However, the primary strategy for ongoing mitigation involves structural additions or alterations to existing confinement housing [29]. St-Pierre et al. [10] classified these housing-based heat abatement strategies into four categories that represent increasing levels of abatement: Minimal < Moderate < High < Intense.

Minimal heat abatement involves a covered structure such as an open barn or shed, which shields animals from incoming radiation while providing natural ventilation, plus ample access to clean, cool water. In Florida, shaded cows experienced 6% to 10% higher milk production, and lower rectal temperatures and respiration rates than their non-shaded counterparts [42,43]. The Minimal heat abatement can be easily implemented and is economical in many locations [44]. Moderate abatement incorporates forced ventilation (fan systems or tunnel-ventilation) into housing structures to increase convective heat loss through improved air movement and exchange [45]. Tunnel ventilation is implemented by running large exhaust fans at high flow rates at one endwall of a barn, thereby creating longitudinal air movement along the barn. Moderate abatement was shown in New York and Ohio to lower indoor ambient temperature by 0.4°C relative to natural ventilation [46]. Holstein dairy cows experienced 50% slower rectal temperature rise and 14 breaths/min slower respiratory rate in northern Israel under moderate abatement relative to natural ventilation [47]. High heat abatement introduces water sprinkling to increase the cooling effect of forced ventilation. Studies conducted at different locations and under variable environmental conditions generally showed that High heat abatement yielded more comfortable environmental conditions for cows and increased milk production as compared to unmitigated, outside conditions [48–51]. In Florida, Bucklin et al. [27] observed a ~6-unit lower THI under High heat abatement as compared to outdoor THI. In Missouri, Igono et al. [24] found that milk production under High heat abatement was 2 kg/cow greater than in shade conditions. Milk production during summer 1988 was 15.9% greater in Kentucky under high heat abatement relative to shading alone [52]. Intense heat abatement involves air conditioning or high pressure evaporative cooling and adoption is generally inhibited by high capital expense [27]. A study conducted by Hahn and Osburn [53] indicated that U.S. southern regions (West, Southwest, South, Ohio Valley, and Southeast) would be the most economically suitable areas for intense heat abatement. Previous studies demonstrated the efficiency of Intense heat abatement. In Thailand, Khongdee et al. [54] found that evaporative cooling decreased THI levels by 3 units relative to no evaporative cooling, and that the physiological impacts of heat stress on Thai Friesian crossbred cows raised under evaporative cooling were generally dampened, leading to 1 kg/cow/day greater milk production. In northern Mississippi, Smith et al. [55] found that mean peak daytime barn THI under Intensive cooling was ~3 units lower relative to Moderate or High heat abatement. Smith et al. [55] found that Holstein dairy cows had 3 to 5 kg/cow/day greater milk yield under Intense cooling than under High heat cooling. Holstein cows raised in a hot and dry climate condition (Saudi Arabia) under Intense heat abatement had improved pregnancy rates compared to those raised under High heat abatement (35% vs. 23%), and greater milk production (0.98 kg /cow/day) [56]. Collier et al. [57] found that Intense heat abatement improved barn environmental conditions and cow physiological functions (respiration rate, rectal temperature) in arid environments, but its effects depend on system design (fans quantity, position, and speed; rate of misting water injection).

St-Pierre et al. [10] estimated that dairy cows housed with Minimal heat abatement experience daily milk production losses that can be modeled using Eq (3).

$$MILKloss_{d}=0.0695*{\left(THImax_{d}-THIthresh\right)}^{2}*D_{d}$$ (3)

where MILKloss_d_ represents the daily milk production loss per cow, THIthresh is the THI threshold, i.e., the upper limit of the homeothermic maintenance zone, which varies by animal. For dairy cows, THIthresh is 64 to 76 [58]. We selected the value proposed by Johnson et al. [59] and used by St-Pierre et al. [10] (THIthresh = 70). D_d_ is the daily ratio of the number of hours during which THI is above THIthresh to the total hours in day d. We used a sine model approximation to estimate D_d_ (S2 File). St-Pierre et al. [10] developed mathematical models for Moderate, High, and Intense heat abatements [Eqs (4), (5) and (6), respectively] based on literature data and Monte Carlo simulations to estimate the potential degree to which THImax_d_ and THImin_d_ are lowered each day (ΔTHI_d_) with the implementation of each cooling level.

$$\text{Moderate cooling:}\;\;\;\;\Delta THI_{d}=-11.06+\left(0.25T_{d}\right)+\left(0.02Rh_{d}\right)$$ (4)

$$\text{High cooling:}\;\;\;\;\Delta THI_{d}=-17.6+\left(0.36T_{d}\right)+\left(0.04Rh_{d}\right)$$ (5)

$$\text{Intense cooling:}\;\;\;\;\Delta THI_{\;d}=-11.7-\left(0.16T_{d}\right)+\left(0.18Rh_{d}\right)$$ (6)

where T_d_ is the minimum or maximum daily temperature (°C) and Rh_d_ is the average daily relative humidity (%). To estimate milk loss under each abatement, we added the appropriate ΔTHI_d_ [Eqs (4), (5) and (6)] to THImax_d_ and THImin_d_ [Eqs (1) and (2)] and then recalculated D_d_ and MILKloss_d._ We aggregated the estimated daily MILKloss_d_ per cow to annual milk production loss.

### Cost-benefit ratio analysis

To conduct an economic comparison of the heat abatement methods, we estimated the marginal milk production present income (benefits) and the present costs of implementation of each heat abatement (costs). The marginal milk production income was the revenue from the additional milk produced using a heat abatement method above the base production from Minimal heat abatement. We used the 1998–2000 market year average milk price of $0.31 /kg [60] for Late-20^th^ century and the 2008–2014 market year average milk price of $0.41 /kg [60] for the 21^st^ century. For each heat abatement, we calculated a cost-benefit ratio. The analysis was conducted separately for each selected location and averaged within climatic regions. Both the benefits and costs were prorated across 20 years to match the lifespan of a barn [61] using a 3% real discount rate (5% discount rate and 2% average inflation rate). A heat abatement was considered economical if the cost-benefit ratio was equal to or less than 1. We assessed the sensitivity of the cost-benefit ratios to the different costs involved in their calculation. We also assessed the effects of milk price on the cost-benefit ratios. Table 1 summarizes the cost specifications of heat abatement evaluation. Further assumptions and calculation details are provided in S3 File.

**Table 1:** Mean milk prices and heat abatement cost estimation data.

Category	Item	Unit	Value	Reference
**Income**				
	1998 to 2000 average milk price	$ /kg	0.31	[60]
	2008 to 2014 average milk price	$ /kg	0.41	[60]
**Moderate heat abatement**				
	Naturally ventilated freestall barn	$/m2	215	[62]
	Fan capital cost (90 cm diameter fan)	$ /each	800	[62]
	Number of fans per stall	Fan/stall	0.2	[63]
	Fan maintenance	$/year	30	[64]
	Fan wattage	kW	0.5	Vendor literature
	Fan residual value	%	10	Vendor literature
	Fan lifespan	year	7	[63]
**High heat abatement (sprinkler)**				
	Purchase and installation	$/stall	19.5	[65,66]
	Water consumption	L/day/stall	64	[65,66]
	Lifespan	year	5	[65,66]
	Residual value	$/stall	0.73	[65,66]
**Intense heat abatement (evaporative cooling)**				
	Purchase and installation	$/stall	130	Vendor literature
	Lifespan	year	10	Vendor literature
	Residual value	%	0	Vendor literature
	Pump wattage	kW/stall	0.047	Vendor literature
	Pad lifespan	year	3	Vendor literature
	Pad replacement	$/stall	51.25	Vendor literature
**Other**				
	Electricity (2012 to 2016)	$/kWh	0.1042	[67]
	Water	$/1000 L	0.21	[63]
	Discount rate (2007 to 2017 80^th^ percentile of secondary credit rate)	%	5	[68]
	Inflation rate	%	2	[69]

### Indices calculation and data analysis

We performed all analyses in the R environment [70] using the Agricolae package [71]. Total annual milk production loss per cow was calculated for each combination of location, GCM, and abatement level. We calculated annual heat stress frequency (HSF) as the annual count of days with mean THI (daily average of maximum and minimum THI) > 70, for each combination of location and GCM. Coefficients of determination (R^2^) and percent biases (PBIAS) [72] were used to compare the THI and milk production loss estimates to calculated values reported in the literature. We used a one-way ANOVA followed by a post-hoc Tukey’s Honest Significant Difference test to compare milk production losses between abatement levels by time-frame at each location and within each climatic region. Although the analytical methods used in this study appeared straight forward and deterministic, GCMs projections have inherent uncertainties. As such, we used nine different models to account for these uncertainties, and therefore we feel that the use of ANOVA to differentiate between summaries was justified.

## Results and discussion

### THI and milk production loss estimation assessment

To evaluate our THI estimation method, we compared the average minimum and maximum THI in July from 1950 to 2000 to values from St-Pierre et al. [10], who estimated average minimum and maximum THI in July over a much longer period from 1871 to 2001. Mean July minimum and maximum THI estimates were generally within 14% and 7%, respectively, of values reported by St-Pierre et al. [10] (R^2^ = 0.74, R^2^ = 0.65, respectively; S2A and S2B Fig). Difference in THI estimation methods (this study used the equation of Yousef [40] and St-Pierre et al. [10] used the equation of Ravagnolo [73]) might have contributed to deviations in estimated THI. Our historic estimates of milk production loss per cow (1950 to 1999) generally correlated well with those from Mauger et al. [15] (R^2^ = 0.86, PBIAS = -41%, S2C Fig) and St-Pierre et al. [10] (R^2^ = 0.48, PBIAS = 24%, S2D Fig). Small discrepancies likely resulted from spatial scale differences. While St-Pierre et al. used observed historical state-level weather data, Mauger et al. used gridded data, and this study used site-specific simulated weather data with variable spacing (distance between points). Differences in averaging periods between this study and St-Pierre et al (50 years in this study vs. 130 years in St-Pierre et al.) may have also contributed to discrepancies.

### Late-20^th^ century

#### Heat stress variability

The magnitude of late-20^th^ century heat stress generally increased from North to South (S3A and S3B Fig) in accordance with the July mean minimum and mean maximum THI trend described by St-Pierre et al. [10]. In the northern U.S. (Northwest, Northern Rockies, Upper Midwest, Northeast), mean daily minimum and maximum THI varied between 38.1±0.1 to 50.1±0.1 and 72.4±0.1 to 76.1±0.1, respectively, whereas in the southern U.S. (West, Southwest, South, Ohio Valley, Southeast) mean daily minimum and maximum THI varied between 39.2±0.1 to 63.7±0.0 and 74.1±0.1 to 81.3±0.1. On average, the southern U.S. experienced smaller THI ranges (<27) as compared to the northern U.S. (up to 37) (S3C Fig). HSF also exhibited a geographical trend similar to minimum and maximum THI but incorporated an East to West trend as well (S3D Fig). In the northern regions, HSF varied between 1 and 54 day/year (mean = 22 day/year; 27 day/year in the Northeast region vs. 5 day/year in the Northwest region) whereas the southern U.S. experienced HSF between 3 and 209 day/year (mean = 86 day/year; 140 day/year in the Southeast region vs. 66 day/year in the Southwest region).

Overall, variability in late-20^th^ century heat stress across the 9 U.S. regions was driven by variability in THI and HSF. HSF also accounted for most of the differences in heat stress between locations in the West and East regions, which was mainly due to humidity. Humidity was also an important heat stress driver in the Southeast region as compared to the other regions (high temperature, high humidity). In addition to geographical heat stress variability, larger THI ranges in the North suggested that cows experienced varying degrees of heat stress, giving them periodic opportunities to cool off and thereby lower adverse impacts on milk production [58]. In the south, however, narrower mean daily THI ranges limited the opportunity for cows to cool off and therefore resulted in a much greater need for continuous heat abatement implementation, especially during summer.

#### Milk production losses under minimal abatement

**Fig 2:**
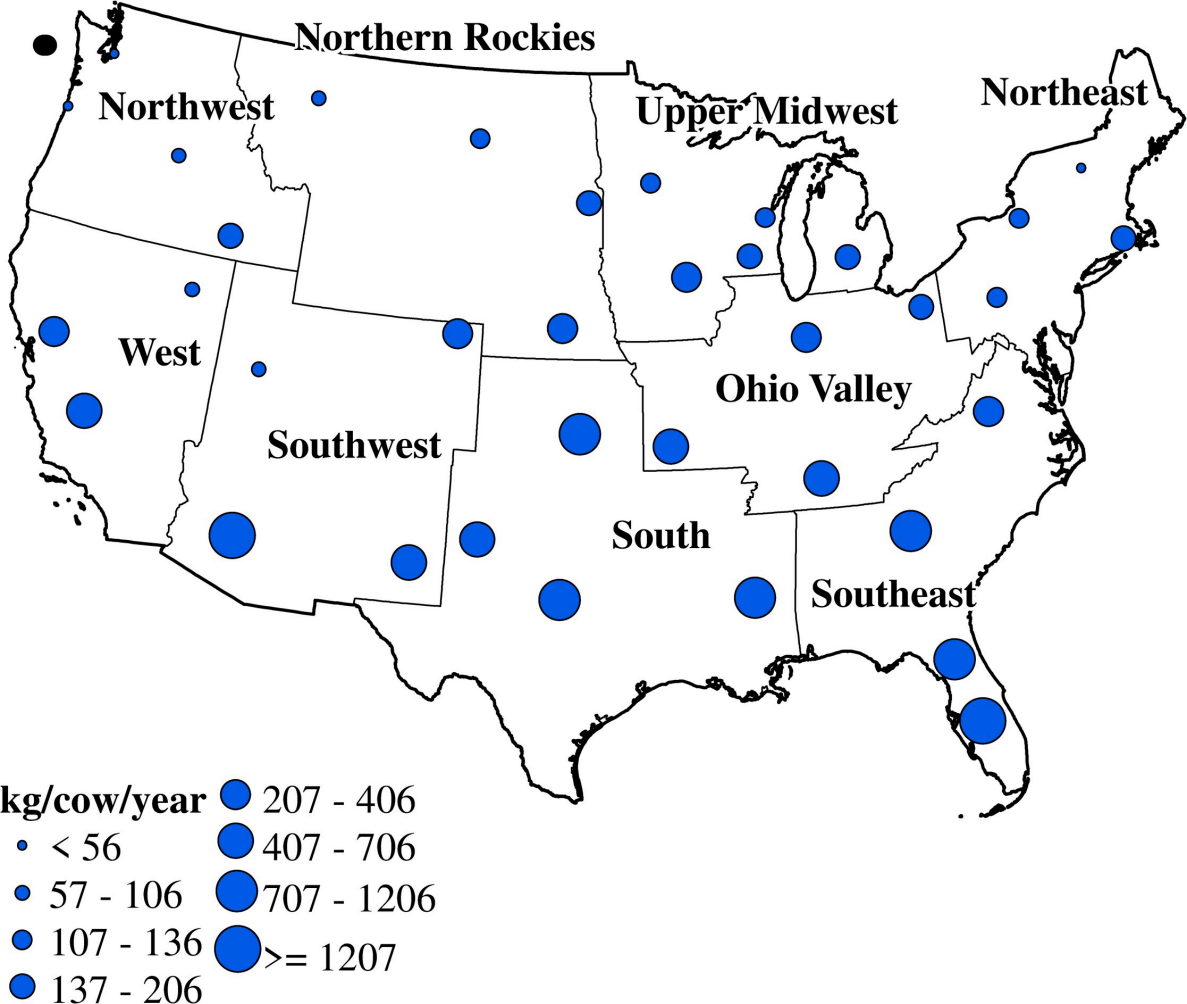
Late-20^th^ century average milk production loss per cow across the U.S. under Minimal heat abatement (Boundary data [31]).

During late-20^th^ century, annual milk production loss per cow under Minimal heat abatement varied largely between climatic regions (p-value < 0.05) and ranged from 6 kg/cow/year in the northern regions to 2,260 kg/cow/year in the southern regions (Fig 2). Mauger et al. [15] and St-Pierre et al. [10] estimated similar trends in mean annual milk production loss per cow for late-20^th^ century (4 to 2,170 kg/cow and 70 to 2070 kg/cow, respectively). The geographic variability of milk production loss per cow was directly related to the geographic variability of temperature, as noted by Mauger et al. [15], as well as variations in THI and HSF across the U.S.

According to the National Animal Health Monitoring System (NAHMS) [74], 83% and 42% of all dairy operations in the U.S. used covered structures and shading, respectively, for lactating cows, with greater use of covered structures in the East than in the West during Summer (80% vs. 75%). Concurrently, 76% and 20% of all lactating cow operations in the U.S. used fans and tunnel ventilation for cooling, respectively, with a larger proportion used in the East. Notably, 25% of dairy operations in the U.S. cool their lactating cows with sprinklers or misters, with the largest use of these systems in the western U.S. [74]. Indeed, the percentage of U.S. dairy operations using covered structures and shading was not restricted to Minimal heat abatement, but most likely applied to the other heat abatements (Moderate, High, and Intense). As such, we speculated that a small percentage of dairy cow operations in the U.S. exclusively used Minimal heat abatement, and that even if milk production losses under Minimal heat abatement were regionally large (as noted in the Southern regions), average milk production losses at the national scale would be tempered.

### 21^st^ century expectations

#### Projected heat stress changes

**Fig 3:**
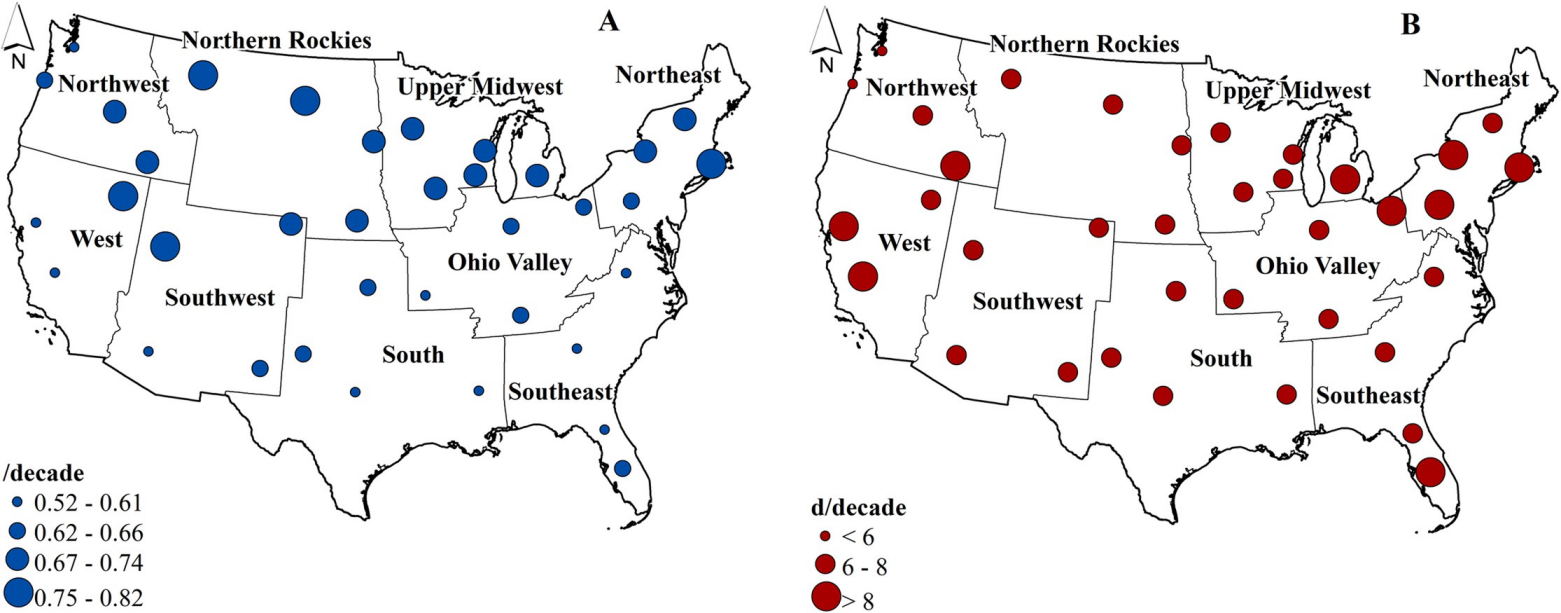
Projected decadal increases in average Temperature Humidity Index (A) and annual Heat Stress Frequency (B) between 2000 to 2100 (Boundary data [31]).

Dairy cows are expected to experience increased discomfort throughout the 21^st^ century, under the RCP 8.5 scenario, as heat stress increases over time in response to rising temperatures. Average annual THI is generally expected to increase at faster rates in the northern regions as compared to southern regions (0.82 /decade in the North and 0.52 /decade in the South, Fig 3A). Mean annual minimum THI is generally expected to increase faster than mean annual maximum THI between 2000 and 2100, and the rapid increase of minimum THI will expectedly lead to a reduction of the diurnal THI range, which again suggests that opportunities for cows to cool off after a daily period of heat stress will likely diminish. Overall, annual HSF (Fig 3B) across the U.S. is likely to increase at a rate of 5 to 11 day/decade, with the fastest increase in the West, Northeast, and Southeast regions. Spring and Fall will likely see HSF increases mainly in the southern regions (S4 Fig), but the reverse trend is projected for summer as HSF is projected to increase by 5 to 8 day/decade in the northern regions as compared to 0 to 4 day/decade in the southern regions.

#### Projected changes in milk production losses under minimal heat abatement

**Fig 4:**
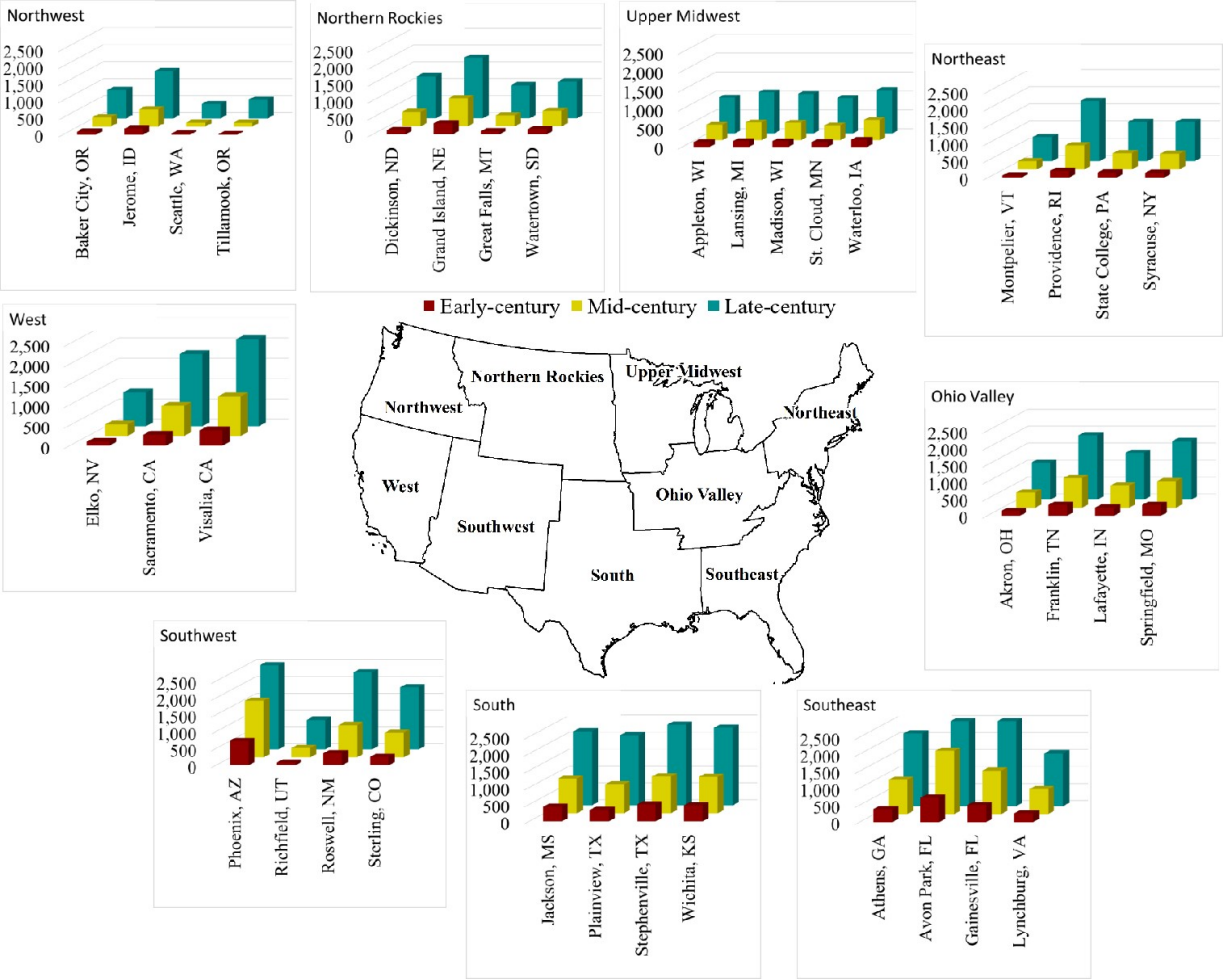
Projected mean annual milk production loss per cow (kg/cow/year) under representative concentration pathway 8.5 with minimal heat abatement by location and period (map boundary data [31]).

U.S. milk production loss under Minimal heat abatement is projected to increase throughout the 21^st^ century, under the RCP8.5 scenario, at a mean rate of 174±7 kg/cow/decade (Fig 4), with the fastest increase in the Southeast region (284 kg/cow/decade) and the slowest increase in the Northwest region (79 kg/cow/decade). These increases in milk production losses are in line with previous studies by Key and Sneering [16] and Mauger et al. [15], which collectively highlight the Southeast region as the most vulnerable to heat stress impacts. According to data reported by the U.S. Department of Agriculture-National Agriculture Statistics Service [75], the average dairy cow produced 9,670 kg/year of milk between 2008 and 2014 across the U.S. Using this mean milk production for extrapolation, U.S. mean annual milk production per cow would decline by 11.6% on average (1.2% to 40.8% depending on the location) by mid-21^st^ century, and by 20.8% on average (4.6% to 60.2%) by late-21^st^ century, due to heat stress. Notably, this ignores potential changes in adaptation due to genetic and management improvements. Larger dairy cattle breeds with high milk production will be disproportionately affected by heat stress with greater milk losses [28]. Our study estimated milk losses from 550 to 650 kg dairy cattle which is smaller than a large Holstein (> 750 kg). High milk producing cows are more susceptible to heat stress than lower yielding breeds of dairy cattle [76]. Predicted future losses are therefore conservative if dairy cattle weight for the U.S. increases with the expansion of confinement strategies [77], along with an increase in milk yield. Projected milk production losses could amount to large income losses for dairy producers. Certainly, there is a strong incentive to implement heat stress abatement measures throughout the 21^st^ century that will support the U.S. dairy industry as climate change progresses. The projected losses in milk production per cow in the Southwest and West regions are of particular concern as these regions recently (2000 to 2016) expanded their milk production by 0.8% and 6.7%, respectively [78], and further expansions are expected in the coming decades.

### Heat abatement effects on milk production loss throughout the 21^st^ century

#### Milk production loss per cow

**Fig 5:**
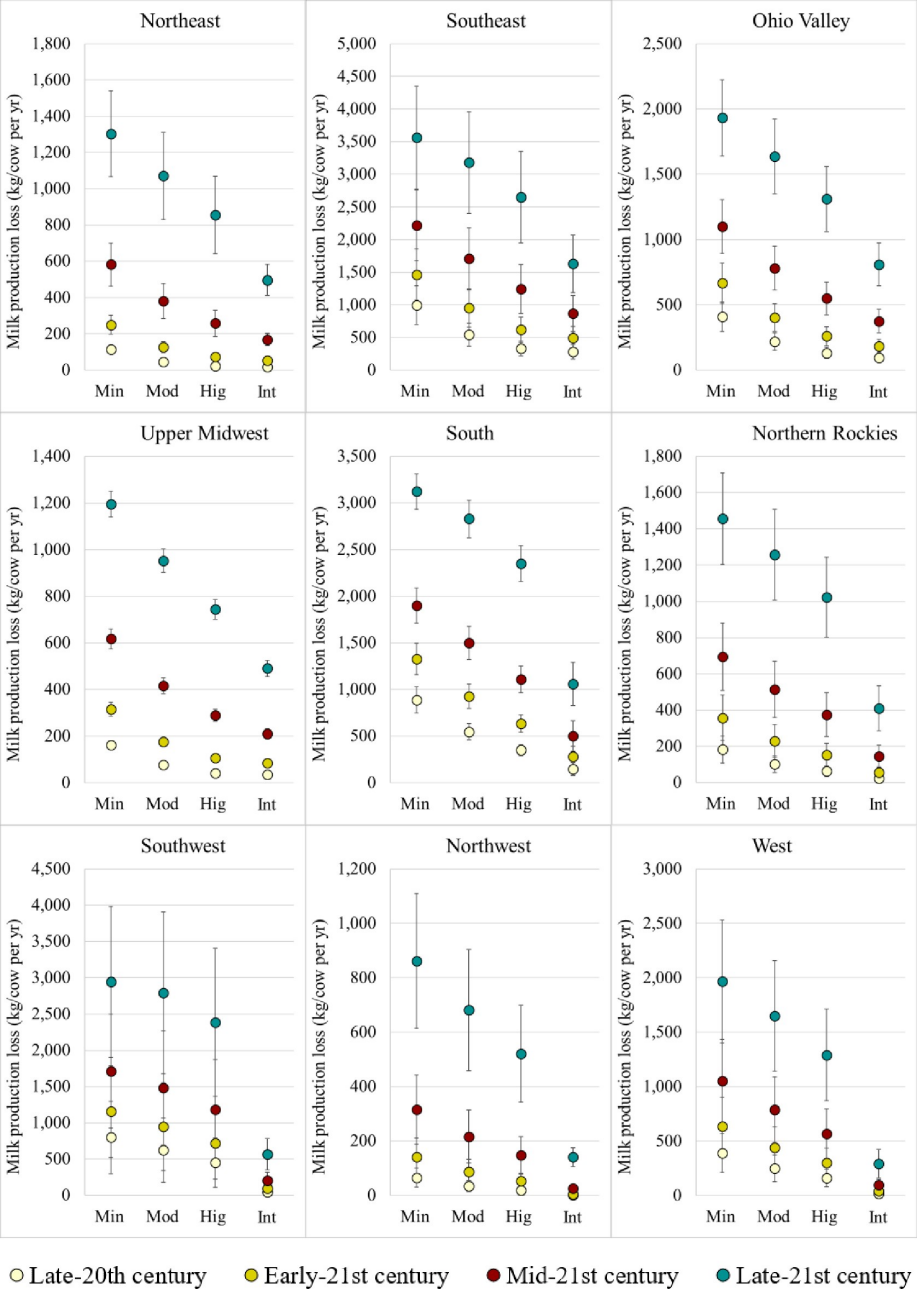
Mean annual milk production loss (with error bars) by climate region, period, and heat abatement (Min: Minimal; Mod: Moderate; Hig: High; Int: Intense).

As expected, the severity of milk production losses is likely to decrease across all regions with the implementation of subsequently stronger levels of heat abatement throughout the 21^st^ century (Fig 5) that enable reductions in mean daily THI (especially daily maximum THI) and HSF. Reductions in mean daily THI will potentially lessen the impacts of heat stress on lactating dairy cows, allowing them to better utilize the energy they obtain from feeds, thus improving feeding efficiency [8,9]. In addition, feed consumption will likely increase as a result of lower apparent heat stress [10], as cows are expected to spend less time dissipating heat (increased water consumption, increased breathing rate). On average, under Moderate, High, and Intense heat abatements, annual milk production per cow is expected to increase by 3%, 4%, and 6% during early-21^st^ century, 3%, 6%, and 11% during mid-21^st^ century, and 3%, 8%, and 21% during late-21^st^ century relative to Minimal heat abatement, respectively. Therefore, the effectiveness of the High and Intense heat abatement will most likely increase as the 21^st^ century progresses. However, average milk production per cow per year has been increasing for many years [75]. If this increasing trend holds, the higher producing cows may become more sensitive to heat stress. Therefore, stronger heat abatement appears necessary in the future to maintain dairy cows in physiologically thermoneutral states.

Results demonstrated that all four heat abatements may provide significant heat stress relief, hence lowering milk production losses per cow in all regions. Ultimately, Intense heat abatement would yield the largest milk production across all regions. However, Intense heat abatement would be more expensive to implement, and may not return much greater milk production than High heat abatement. This is particularly true for some regions (Northeast and Upper Midwest) in early-21^st^ century, as differences in milk production loss per cow between the two heat abatements were generally insignificant (S2 Table). A similar pattern was estimated for mid-21^st^ century again between High and Intense heat abatements at some locations in the Northeast (Montpelier, VT and State College, PA) and Upper Midwest (Lansing, MI), and between Moderate and High heat abatements in the Northwest (Tillamook, OR). In Phoenix (Southwest region), significant effects on milk production were estimated only under High and Intense heat abatement. In late-21^st^ century, heat abatements stronger than Minimal were not effective at Tillamook, OR, and Intense heat abatement was not much more effective than High heat abatement.

#### Economic feasibility of abatement impacts throughout the 21^st^ century

Heat abatements generally lowered the impacts of increasing heat stress and returned greater milk production across the U.S. regions during the 21^st^ century, under the RCP 8.5 scenario. Their economic feasibility was generally supported, nonetheless some regions showed no expectation of profits during some periods of the 21^st^ century. Profits in this paper were the marginal net returns related to the implementation of Moderate, High, and Intense heat abatements relative to the Minimal heat abatement (base scenario), and did not refer to the earnings of the entire milk production activity.

**Fig 6:**
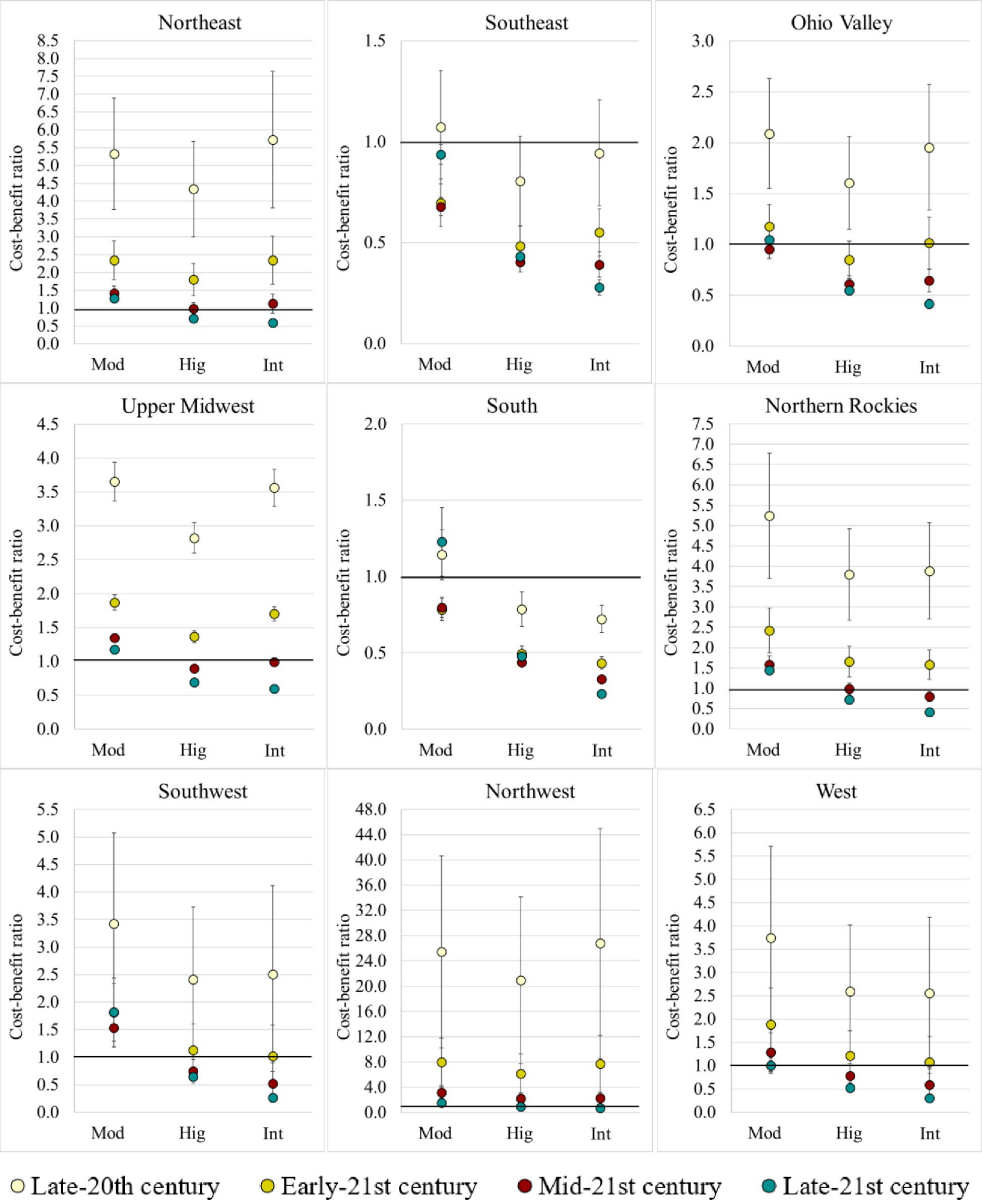
Mean cost-benefit ratios (with error bars) associated with heat abatements by region and period (Mod: Moderate; Hig: High; Int: Intense).

Mean milk production losses under early-21^st^ century weather conditions in the Northeast, Upper Midwest, Northern Rockies, and Northwest regions were at first relatively low under Minimal heat abatement (<400 kg/cow/year, Fig 5), and there was not incentive for heat abatement. Therefore, the implementation of heat abatement was not economically supported regardless of the abatement level (cost-benefit ratio >1, Fig 6). In the Southeast and South regions where mean milk production losses under Minimal abatement approached 1,500 kg/cow/year, the cost-benefit ratios were <1 under the Moderate, High, and Intense heat abatements, yielding $60±30 /cow/year, $150±50 /cow/year, and $160±60 /cow/year in profit for the Southeast, and $30±10 /cow/year, $110±20 /cow/year, and $190±30 /cow/year in profit for the South, respectively. Cost-benefit ratios <1 were also estimated for the Ohio Valley under High and Intense heat abatements (estimated annual profits were $60±30 /cow for each) and for the Southwest region under Intense heat abatements (estimated annual profit was $200±170 /cow). The estimated mean annual profit for the Southwest region under High heat abatement was $40±40 /cow, but the mean cost-benefit ratio was weighted toward more than one by the ratio determined for Richfield, UT. Similarly, the West region would experience annual profits under High and Intense heat abatements ($20±30 /cow and $60±70 /cow, respectively) although the mean cost-benefit ratios indicated otherwise due to elevated ratios for Elko, NV. Compared to the other locations in their respective regions, Richfield, UT and Elko, NV experienced lower maximum and minimum THI (75.2±0.1 to 76.0±0.1 and 44.1±0.1 to 45.1±0.1, respectively, S3 Table) as well as HSF (~13 day/year, S3 Table), resulting in lower milk production losses and lower requirements for abatement.

During mid-21^st^ century, mean base milk production losses (under Minimal heat abatement) were relatively high in the Southeast, Ohio Valley, and South regions (>1000 kg/cow/year), and significant production increases were projected under Moderate, High, and Intense heat abatements, making abatement implementation economically feasible at these three levels. Between the three heat abatements, these regions could receive annually $50±10 /cow to $270±70 /cow, $10±10 /cow to $90±40 /cow, and $30±10 /cow to $290±30 /cow in profit, respectively. High and Intense heat abatements were also economical options for the Northeast, Upper Midwest, Northern Rockies, Southwest, and West regions, as the average milk production increases at these levels were significant enough to compensate for their costs of implementation. Across these regions, the estimated annual profits ranged from $10±10 /cow to $70±40 /cow and $0±10 /cow to $330±200 /cow for the High and Intense heat abatements, respectively. However, High heat abatement appeared to deliver the largest annual profits in the Northeast and Upper regions ($10±10 /cow/year for High abatement vs. $0±20 /cow/year for Intense abatement). All the regions except Southeast and West experienced profitability under High and Intense heat abatements during late-21^st^ century (estimated annual profits were $20±20 /cow to $160±20 /cow and $90±10 /cow to $590±250 /cow for High and Intense heat abatements, respectively). The Southeast and West regions experienced profitability under the Moderate heat abatement, with annual profits amounting to $10±10 /cow. The lack of profit under Moderate heat abatement indicated that milk losses under late-21^st^ century weather conditions would not be amenable to beneficial milk production across most regions in the U.S. if at least High heat abatement is not implemented (except Southeast and West regions). Ultimately, Intense heat abatement would deliver the largest profits.

Overall, basic trends indicated that higher levels of heat abatement (High and Intense) are expected to become increasingly cost-effective later in the century for all regions, under the higher scenario, even the northerly locales that seem to be buffered against heat stress in the early- and mid-21^st^ century. As the 21^st^ century progresses, dairy producers will likely need to switch to stronger heat abatement to mitigate the effects of heat stress on milk production, especially in late-21^st^ century. The Northeast, Upper Midwest, and Northern Rockies would not benefit from heat abatement until mid-21^st^ century, during which implementing High heat abatement would be most cost-effective. The Northwest region would benefit from Intense heat abatement only during late-21^st^ century.

Cost-benefit ratio sensitivity analyses showed that annual profits could be positively affected by cost differentials between naturally and mechanically ventilated barns, fan installation and operation costs, discount rates, and inversely affected by milk prices (S5, S6, S7 and S8 Figs). Of the 9 variables we explored in the sensitivity analyses, barn cost differences had by far the largest influence on annual profits. Therefore, for dairy producers, lowering capital costs of mechanically ventilated barns would be critical to determining the economic viability of heat abatement implementation at the farm level.

### Limitations

Expert knowledge suggests that extended heat stress events have lasting impacts on cow welfare, and by extension, a greater impact on productivity. In one particular study conducted under controlled conditions in Brazil, Ferrazza et al. [17] estimated that cows exposed to THIs greater than 75 had consistently lower dry matter intake (DMI) after a period of 3 days. On average across the U.S., the average number of consecutive days during which mean daily THI stays above the threshold THI (70) is expected to rise from 7 in late-20^th^ century to 13 in late-21^st^ century, under the higher scenario. If we speculate that the number of consecutive days with THI above 75 will increase as well, adverse effects on DMI of dairy cows, and consequently on milk production, would clearly be expected. In this study, we assumed that heat abatement implementation reduced the daily degree of THI experienced by dairy cows. However, if the resultant daily THI stays above 75 for more than a 3-day period as suggested by the Ferrazza et al., there could be long-term lasting impacts on milk production, hence on the balance between costs and benefits.

In addition, from a farm herd point of view, even if individual cow milk yields could benefit from heat abatements as estimated here, other factors that may be related to climate change, including change in spatio-temporal insect infestation patterns and change in feed crops availability and quality [19,79], increased culling and death rates, and reduced reproduction rates and feed efficiency [25,28,63,80] could represent hurdles for dairy producers, especially in areas with high mean daily THI (southern regions). Heat stress has a significant adverse impact on the physiologic functions of dry cows, and milk production could benefit from dry period cooling [48,51,81]. Therefore, to enhance the effects of heat abatement at the farm level, dairy producers should consider addressing heat stress impacts not only for lactating cows, but also at other stages of production (calf, heifer, and dry cow). Adoption of heat abatement at all stages of production could alter the cost-benefit ratios estimated in this study.

## Summary and conclusion

This study illustrates the use of deterministic models along with downscaled climate projections in assessing the impacts of heat stress abatement on milk production at local and regional scales. Under the RCP 8.5, downscaled projections of nine CMIP5 global climate models were used to estimate annual milk production loss per cow across nine climatic regions of the U.S., as well as to evaluate the technical and economic effectiveness of four subsequently stronger heat abatement strategies–Minimal, Moderate, High, and Intense–throughout the 21^st^ century.

Findings from the study showed that annual mean THI, a key indicator of dairy cow heat stress, increased nationally, with the fastest rates of increase in the northern U.S. Heat stress frequency (number of days with mean THI>70) also increased, especially in the West, Northeast, and Southeast regions. Annual milk production loss per cow were greatest in the West, Southwest, South, and Southeast regions, making these regions the most vulnerable to climate change for dairy production. Therefore, stronger heat abatement methods are needed across the southern U.S. early in the century, with increasingly intense heat abatement involving air cooling needed in most regions toward the end of the 21^st^ century.

Examining heat abatement strategies in response to climate change, as was done in this study, advances our understanding of climate adaptation strategies for different dairy regions in the U.S., as well as their cost-effectiveness. In light of the need for High, and in some cases, Intense heat abatement in late-21^st^ century, future studies of this sort should consider the full animal lifecycle effects of adopting such intense heat abatement, especially strategies that involve energy-intensive solutions like indoor air conditioning. Future studies could also consider investigating the economic effectiveness of new housing-based cooling technologies (e.g. heat exchanger beds) in association with herd management techniques (crowding avoidance, reduced time in hot holding locations), and diet and genetic manipulation, which may contribute further benefit in heat abatement.

## Supporting information

S1 Fig Predicted vs. observed dew-point temperatures for 2000–2010. State College, PA, monthly (A); Plainview, TX, daily (B).
(TIF)

S2 Fig Predicted vs. St-Pierre et al. (2003) average July minimum (A) and maximum (B) Temperature Humidity Index; Predicted vs. Mauger et al. (2015) annual milk production loss (kg/cow/year) (C); Predicted vs. St-Pierre et al. (2003) annual milk production loss (kg/cow/year) (D).
(TIF)

S3 Fig Late-20^th^ century annual mean Temperature Humidity Index (THI) minimum (A), maximum (B), and range (C), and Heat Stress Frequency (HSF) across the U.S. (Boundary data: https://www.ncdc.noaa.gov/monitoring-references/maps/us-climate-regions.php).
(TIF)

S4 Fig Expected seasonal changes in heat stress frequency between 2000 and 2100 across the U.S.: Winter (A); Spring (B); Summer (C); Fall (D) (Boundary data: https://www.ncdc.noaa.gov/monitoring-references/maps/us-climate-regions.php).
(TIF)

S5 Fig Sensitivity of the mean cost-benefit ratio to barn cost difference, by period and region. (TIF)

S6 Fig Sensitivity of the mean cost-benefit ratio to fan installation and operation costs, by period and region. (TIF)

S7 Fig Sensitivity of the mean cost-benefit ratio to discount rate, by period and region. (TIF)

S8 Fig Sensitivity of the mean cost-benefit ratio to milk price, by period and region. (TIF)

S1 Table Coupled model intercomparison project 5 global climate models used for this study. (PDF)

S2 Table Milk production loss (kg/cow/year) under RCP 8.5, by heat abatement levels. Numbers in the same time frame within the same row followed by the same letter are not significantly different (significance level = 0.5).
(PDF)

S3 Table Annual mean daily summary for maximum and minimum temperature humidity index (THI) and heat stress frequency (HSF) (with standard error of the mean), under RCP 8.5. (PDF)

S4 Table Mean cost-benefit ratios of heat abatement implementation under representative concentration pathway 8.5. Cost-benefit ratios ≤ 1 (shaded) show marginal breakeven or profitability. (PDF)

S5 Table Mean annual net profit (or loss) of heat abatement implementation by regions and time period, under representative concentration Pathway 8.5. (PDF)

S6 Table Late-20^th^ Century milk production losses, net present value, and cost-benefit ratios under different abatements. Two similar letters in the same row following milk production losses show no significant difference (α = 0.05). Cost-benefit ratios ≤ 1 (shaded) show marginal breakeven or profitability.
(PDF)

S1 File Daily dew-point temperature and relative humidity projection. (PDF)

S2 File. E stimation of D_d_ (daily ratio of the number of hours during which Temperature Humidity Index–THI–is above threshold THI–THIthresh–). (PDF)

S3 File Cost-benefit ratio analysis. (PDF)
