# Supplementary figures and images for: Projected heat stress challenges and abatement opportunities for U.S. milk production
Source: PLoS One. 2019 Mar 28;14(3):e0214665. doi: 10.1371/journal.pone.0214665 (PMC6438606; doi:10.1371/journal.pone.0214665)

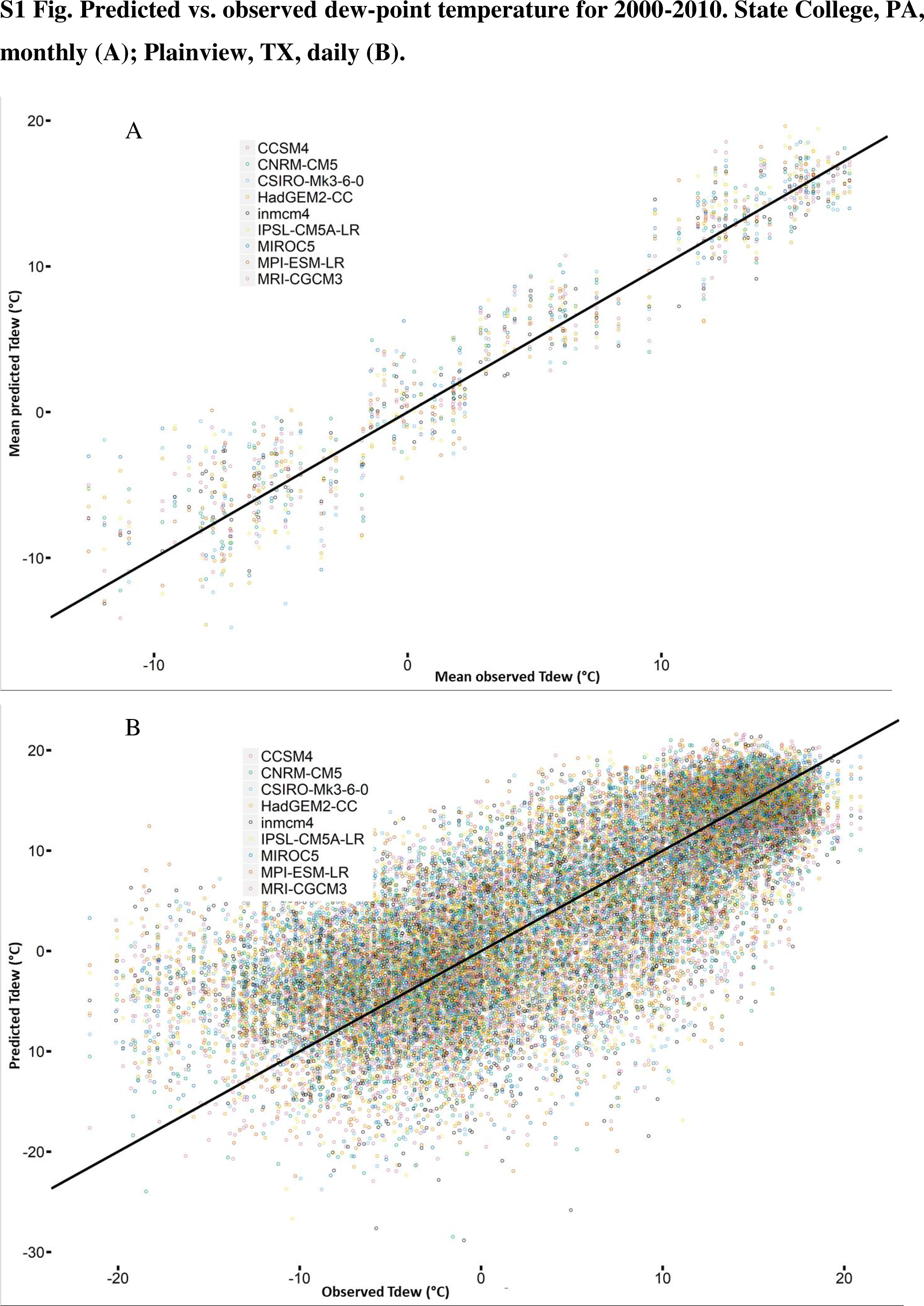

Supplement: S1 Fig — State College, PA, monthly (A); Plainview, TX, daily (B). (TIF) [file pone.0214665.s001.tif]

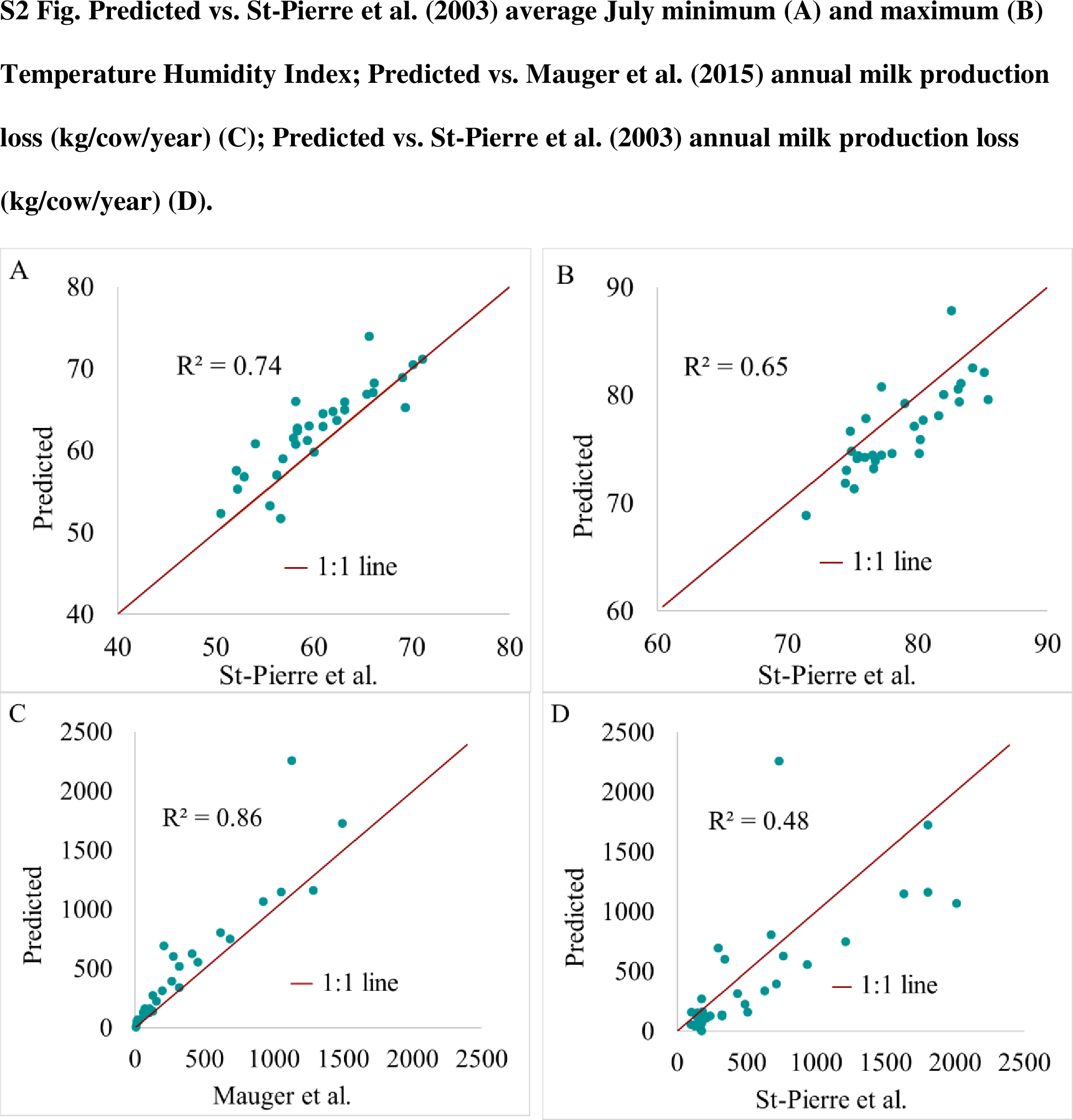

Supplement: S2 Fig — Predicted vs. St-Pierre et al. (2003) average July minimum (A) and maximum (B) Temperature Humidity Index; Predicted vs. Mauger et al. (2015) annual milk production loss (kg/cow/year) (C); Predicted vs. St-Pierre et al. (2003) annual milk production loss (kg/cow/year) (D). (TIF) [file pone.0214665.s002.tif]

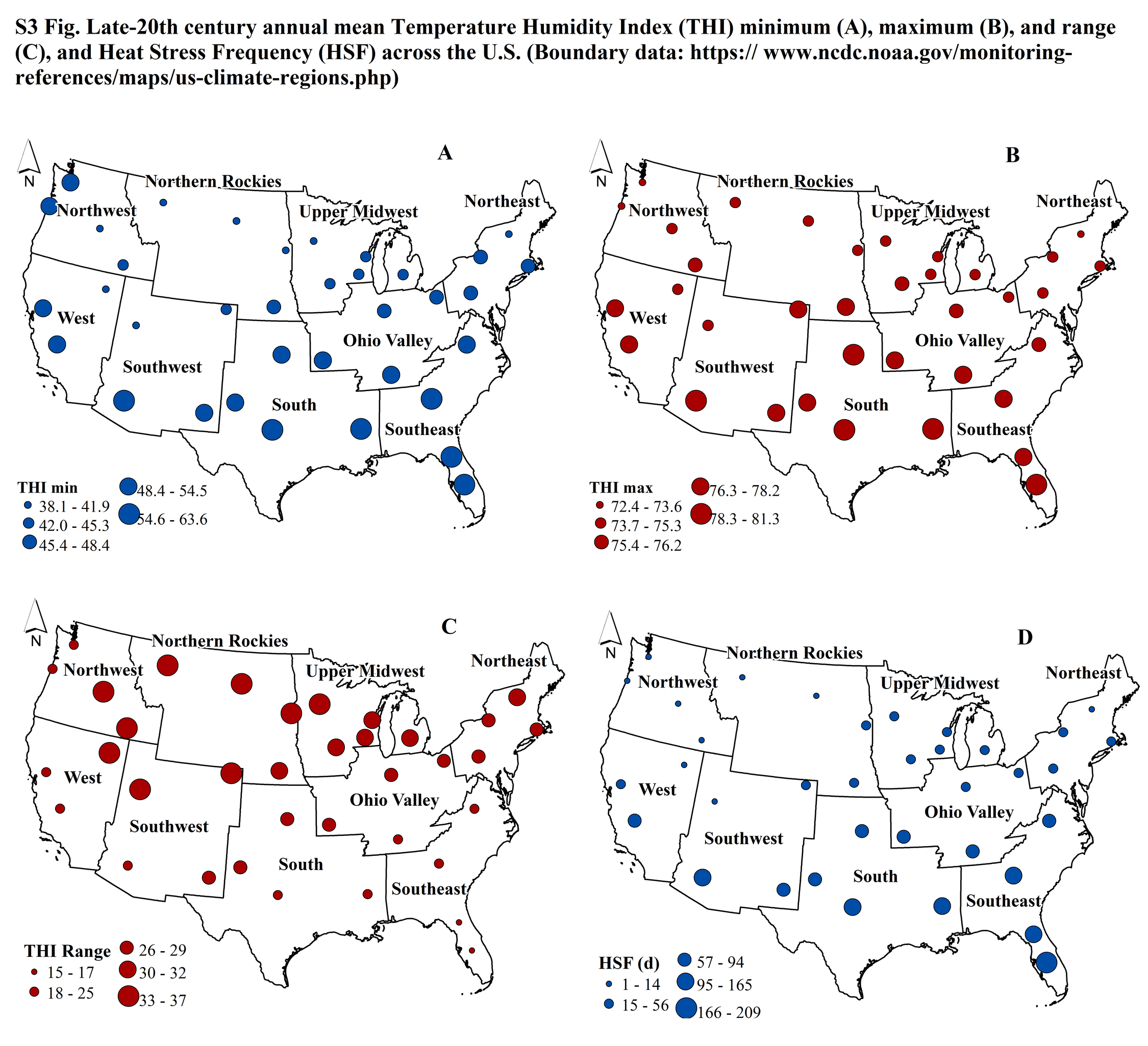

Supplement: S3 Fig — Late-20th century annual mean Temperature Humidity Index (THI) minimum (A), maximum (B), and range (C), and Heat Stress Frequency (HSF) across the U.S. (Boundary data: https://www.ncdc.noaa.gov/monitoring-references/maps/us-climate-regions.php). (TIF) [file pone.0214665.s003.tif]

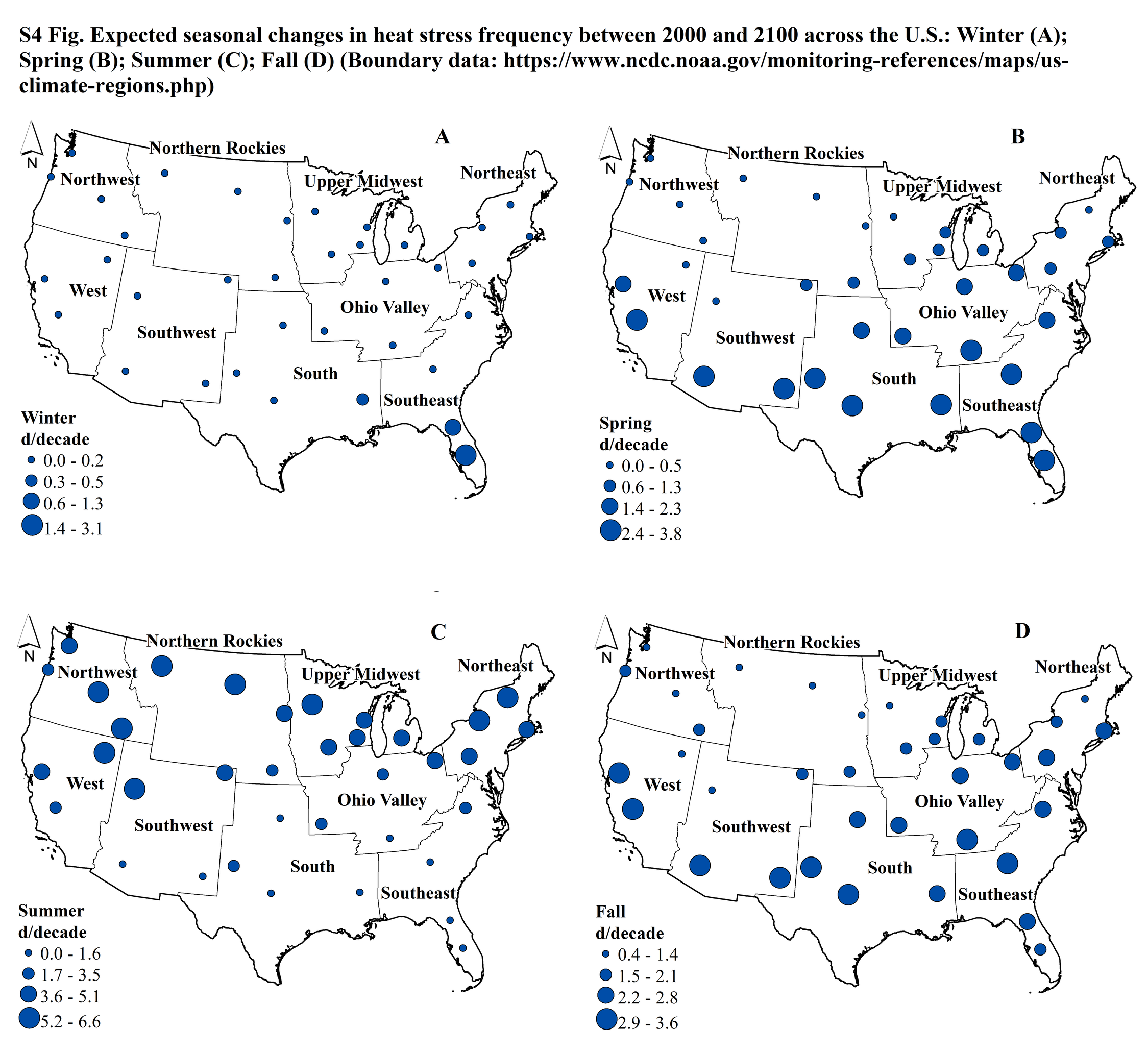

Supplement: S4 Fig — Expected seasonal changes in heat stress frequency between 2000 and 2100 across the U.S.: Winter (A); Spring (B); Summer (C); Fall (D) (Boundary data: https://www.ncdc.noaa.gov/monitoring-references/maps/us-climate-regions.php). (TIF) [file pone.0214665.s004.tif]

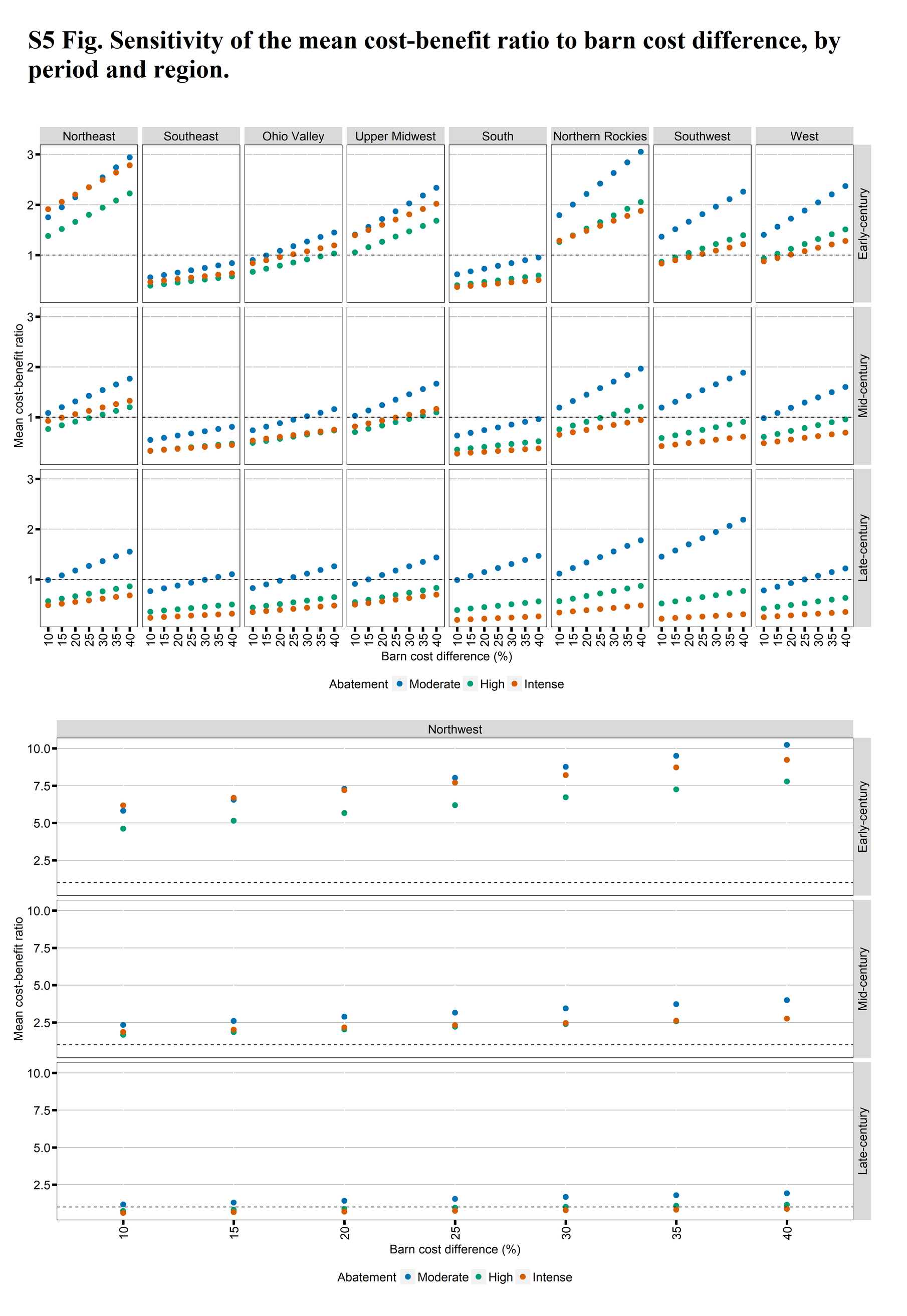

Supplement: S5 Fig — (TIF) [file pone.0214665.s005.tif]

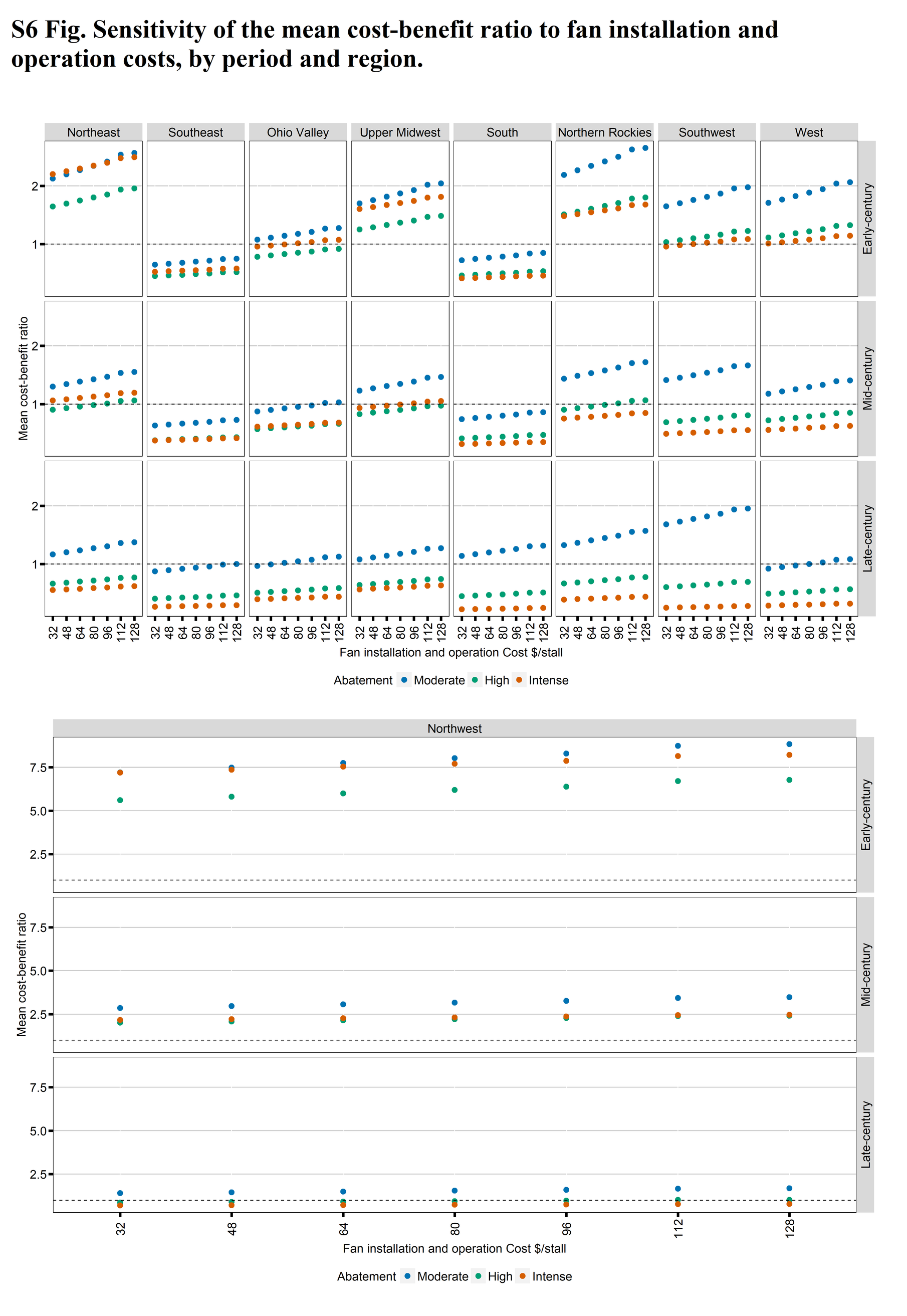

Supplement: S6 Fig — (TIF) [file pone.0214665.s006.tif]

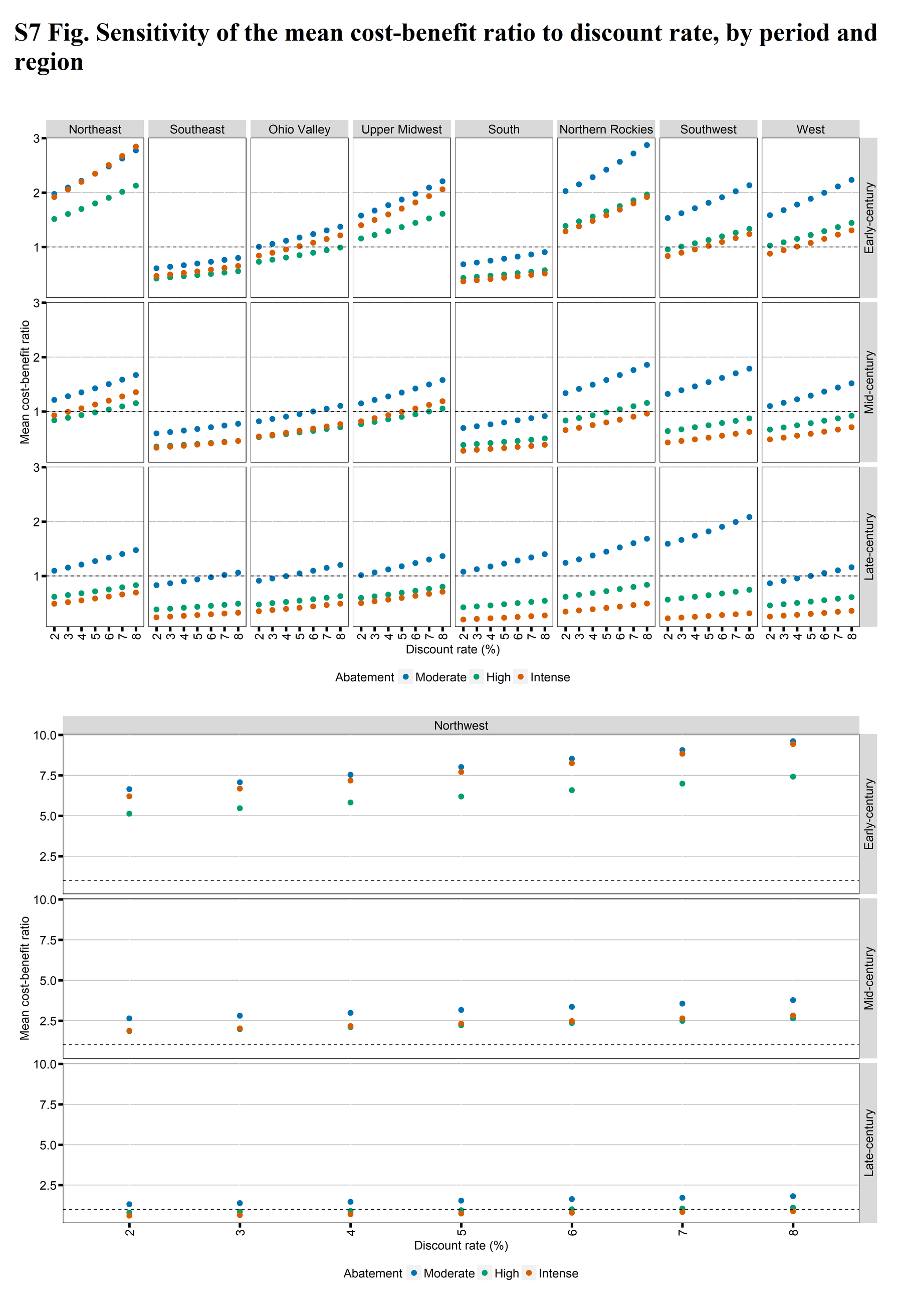

Supplement: S7 Fig — (TIF) [file pone.0214665.s007.tif]

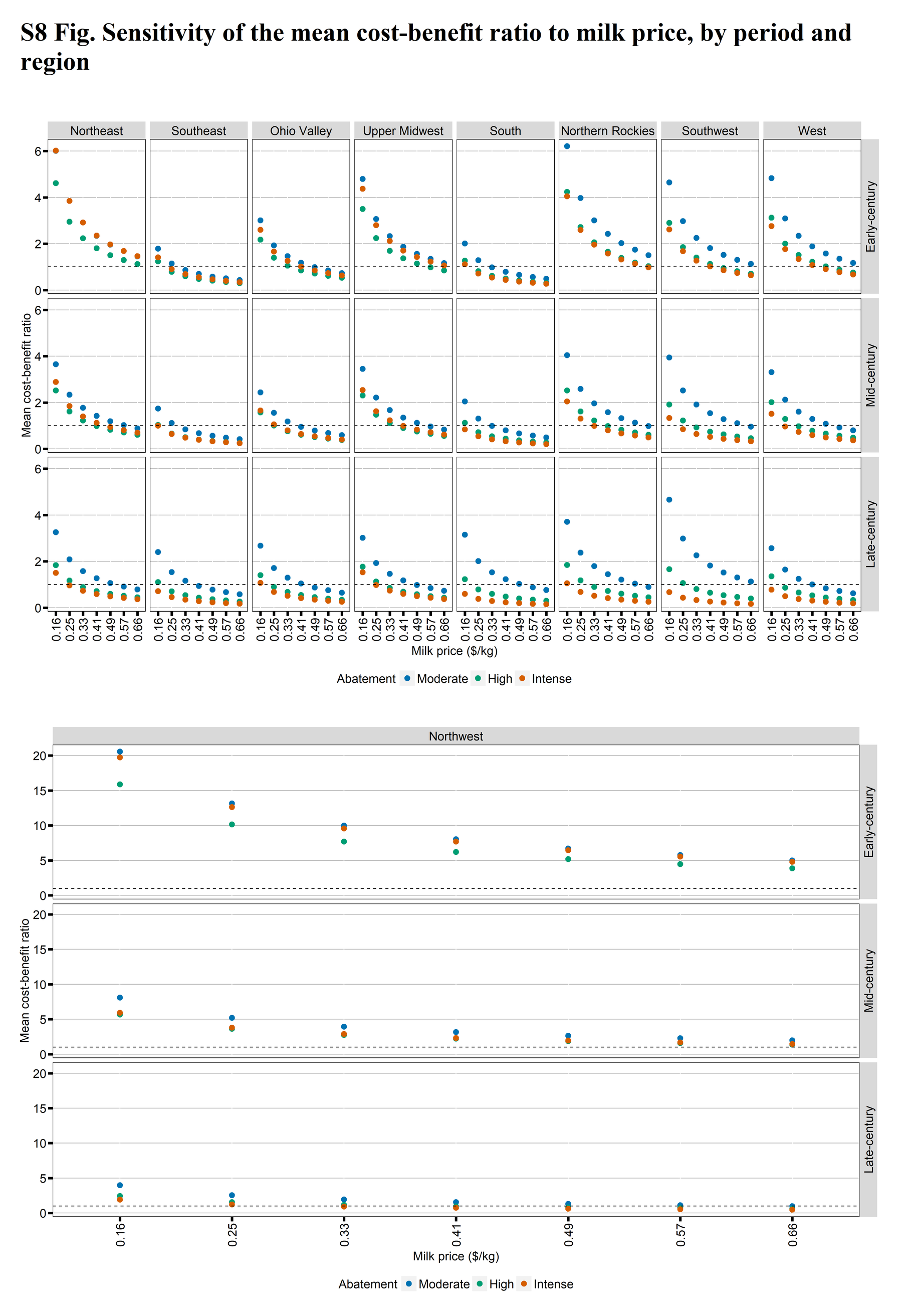

Supplement: S8 Fig — (TIF) [file pone.0214665.s008.tif]
