# Supplementary material for: Projected heat stress challenges and abatement opportunities for U.S. milk production
Source: PLoS One. 2019 Mar 28;14(3):e0214665. doi: 10.1371/journal.pone.0214665 (PMC6438606; doi:10.1371/journal.pone.0214665)
Supplement: S1 Table — (PDF) [file pone.0214665.s009.pdf]

S1 Table. Coupled Model Inter-Comparison Project 5 Global Climate Models used for this study.

**Modeling group**	**Model Name**	**Resolution ***
National Center for Atmospheric Research	CCSM4	0.942° x 1.200°
Centre National de Recherches Meteorologiques	CNRM-CM5	1.401° x 1.406°
Commonwealth Scientific and Industrial Research Organization	CSIRO-Mk3-6-0	1.865° x 1.875°
Met Office Hadley Centre	HadGEM2-CC	1.250° x 1.875°
Institute for Numerical Mathematics	INMCM4	1.500° x 2.000°
Institute Pierre Simon Laplace	IPSL-CM5A-LR	1.895° x 3.750°
Max Planck Institute for Meteorology	MPI-ESM-LR	1.865° x 1.875°
Meteorological Research Institute	MRI-CGCM3	1.121° x 1.125°
Atmosphere and Ocean Research Institute	MIROC5	1.401° x 1.406°

* [1]

Reference

1. ENES. CMIP5 models and grid resolution. In: ENES Model Data and Metadata. 2011. Available from https://portal.enes.org/data/enes-model-data/cmip5/resolution
