# Supplementary material for: Projected heat stress challenges and abatement opportunities for U.S. milk production
Source: PLoS One. 2019 Mar 28;14(3):e0214665. doi: 10.1371/journal.pone.0214665 (PMC6438606; doi:10.1371/journal.pone.0214665)
Supplement: S2 Table — Numbers in the same time frame within the same row followed by the same letter are not significantly different (significance level = 0.5). (PDF) [file pone.0214665.s010.pdf]

S2 Table. Milk production loss (kg/cow/year) under Representative Concentration Pathway 8.5, by heat abatement levels. Numbers in the same time frame within the same row followed by the same letter are not significantly different (significance level = 0.05).

Climatic region	Location	Early-21st Century				Mid-21st Century				Late-21st Century			
		Min*	Mod	Hig	Int	Min	Mod	Hig	Int	Min	Mod	Hig	Int
		kg/cow/year											
Northeast	Montpelier, VT	105a	45b	23c	21c	281a	155b	95c	81c	752a	550b	413c	302c
	Providence, RI	352a	192b	114c	73d	857a	625b	449c	249d	1912a	1714ab	1441b	720c
	State College, PA	277a	134b	75c	62c	603a	368b	238c	176c	1274a	997b	768c	489d
	Syracuse, NY	266a	136b	77c	56c	584a	377b	251c	166d	1271a	1022b	800c	475d
Southeast	Athens, GA	1138a	707b	459c	288d	1760a	1311b	929c	558d	2889a	2548b	2084c	1149d
	Avon Park, FL	2451a	1706b	1133c	925d	3591a	2943b	2218c	1535d	5583a	5204b	4469c	2689d
	Gainesville, FL	1670a	1067b	678c	620c	2442a	1871b	1354c	1073d	3866a	3434b	2868c	1984d
	Lynchburg, VA	604a	329b	200c	133d	1077a	706b	471c	301d	1897a	1534b	1180c	699d
Ohio Valley	Akron, OH	282a	135b	74c	55c	590a	350b	221c	153d	1232a	934b	694c	422d
	Franklin, TN	950a	590b	387c	302d	1500a	1099b	786c	590d	2523a	2202b	1798c	1215d
	Lafayette, IN	557a	335b	213c	168d	964a	690b	492c	354d	1692a	1413b	1135c	731d
	Springfield, MO	877a	554b	365c	213d	1341a	986b	701c	406d	2282a	1996b	1610c	870d
Upper Midwest	Appleton, WI	263a	138b	80c	59c	544a	357b	243c	165d	1087a	853b	656c	407d
	Lansing, MI	308a	166b	99c	89c	623a	410b	281c	224c	1239a	984b	769c	539d
	Madison, WI	321a	180b	110c	90c	631a	427b	298c	223d	1210a	967b	759c	516d
	St. Cloud, MN	259a	144b	89c	61d	523a	348b	240c	160d	1068a	839b	649c	408d
	Waterloo, IA	420a	247b	155c	123c	765a	535b	379c	274d	1375a	1118b	882c	578d
South	Jackson, MS	1583a	1101b	740c	584d	2180a	1730b	1271c	934d	3339a	3008b	2522c	1645d
	Plainview, TX	861a	545b	354c	63d	1378a	976b	677c	154d	2592a	2237b	1771c	518d
	Stephenville, TX	1569a	1092b	747c	241d	2158a	1704b	1251c	461d	3451a	3134b	2592c	1080d
	Wichita, KS	1283a	964b	691c	233d	1886a	1584b	1232c	447d	3098a	2932a	2516b	987c
Northern Rockies	Dickinson, ND	235a	150b	98c	19d	539a	402b	297c	81d	1352a	1200a	1005b	348c
	Grand Island, NE	723a	493b	340c	136d	1231a	964b	727c	309d	2184a	1972a	1649b	729c
	Great Falls, MT	152a	88b	52c	3d	384a	261b	174c	19d	1037a	843b	643c	124d
	Watertown, SD	318a	187b	120c	64d	621a	428b	301c	166d	1246a	1013b	796c	435d
Southwest	Phoenix, AZ	2988a	2726b	2199c	286d	3944a	3784a	3199b	533c	5825a	5930a	5301b	1149c
	Richfield, UT	137a	72b	36c	1d	336a	222b	139c	13d	933a	755b	571c	110d
	Roswell, NM	964a	630b	410c	41d	1560a	1147b	802c	118d	2891a	2575b	2059c	445d
	Sterling, CO	526a	354b	239c	42d	1009a	771b	580c	153d	2119a	1910a	1609b	565c
Northwest	Baker City, OR	153a	90b	53c	4d	338a	228b	151c	20d	900a	699b	523c	119d
	Jerome, ID	333a	217b	136c	7d	668a	487b	340c	31d	1547a	1303b	1018c	163d
	Seattle, WA	57a	26b	13c	2d	140a	74b	43c	9d	445a	291b	197c	57d
	Tillamook, OR	22a	10b	6b	5b	118a	74ab	52b	41b	557a	431ab	344bc	221c
West	Elko, NV	166a	98b	54c	1d	354a	238b	151c	6d	897a	698b	507c	50d

Sacramento, CA	671a	468b	322c	40d	1138a	849b	618c	103d	2173a	1804b	1413c	325d
Visalia, CA	1072a	758b	524c	80d	1664a	1277b	936c	184d	2828a	2443b	1950c	502d

*** Min = minimal; Mod= moderate; Hig = high; Int = intense**
