# Supplementary material for: Projected heat stress challenges and abatement opportunities for U.S. milk production
Source: PLoS One. 2019 Mar 28;14(3):e0214665. doi: 10.1371/journal.pone.0214665 (PMC6438606; doi:10.1371/journal.pone.0214665)
Supplement: S3 Table — (PDF) [file pone.0214665.s011.pdf]

S3 Table. Annual mean daily summary for maximum and minimum Temperature humidity index (THI) and heat stress frequency (HSF) (with standard error of the mean), under RCP 8.5.

Climatic region	Location	Max. THI				Min. THI				Heat stress frequency (day)			
		Past*	Early	Mid	Late	Past	Early	Mid	Late	Past	Early	Mid	Late
Northeast	Montpelier, VT	73.6(0)	74.6(0.1)	76.1(0.1)	78.2(0.2)	41.9(0.1)	43.7(0.1)	46.5(0.1)	50.3(0.2)	9(0)	22(1)	45(1)	76(2)
	Providence, RI	74.9(0.1)	76.3(0.1)	78.5(0.1)	81.3(0.2)	48.2(0.1)	50(0.1)	52.5(0.1)	56(0.1)	38(1)	60(1)	89(1)	119(1)
	State College, PA	74.7(0.1)	76(0.1)	77.6(0.1)	79.6(0.2)	47.1(0.1)	48.8(0.1)	51.1(0.1)	54.2(0.1)	32(1)	54(1)	82(1)	112(2)
	Syracuse, NY	74.7(0.1)	75.9(0.1)	77.6(0.1)	79.7(0.2)	46.1(0.1)	47.9(0.1)	50.5(0.1)	53.9(0.2)	28(1)	48(1)	74(1)	106(2)
Southeast	Athens, GA	77.7(0.1)	78.8(0.1)	80.1(0.1)	82.1(0.1)	55.4(0.1)	56.8(0.1)	58.6(0.1)	61.3(0.1)	114(1)	131(1)	152(1)	178(1)
	Avon park, FL	78.7(0)	80.1(0.1)	81.8(0.1)	84.2(0.1)	63.6(0.1)	65.3(0.1)	67.4(0.1)	70.3(0.1)	209(1)	237(2)	265(2)	292(2)
	Gainesville, FL	77.9(0)	79(0.1)	80.3(0.1)	82.4(0.1)	60.5(0.1)	62.2(0.1)	64.3(0.1)	67.3(0.2)	165(1)	189(1)	214(1)	241(2)
	Lynchburg, VA	76.2(0.1)	77.5(0.1)	79(0.1)	80.8(0.1)	51.1(0.1)	52.6(0.1)	54.6(0.1)	57.3(0.1)	71(1)	93(1)	117(1)	144(1)
Ohio Valley	Akron, OH	74.7(0.1)	75.9(0.1)	77.4(0.1)	79.3(0.2)	47.4(0.1)	49.2(0.1)	51.6(0.1)	54.8(0.2)	34(1)	56(1)	83(1)	115(2)
	Franklin, TN	77.5(0.1)	78.5(0.1)	79.8(0.1)	81.8(0.1)	52.4(0.1)	54(0.1)	56.2(0.1)	59.5(0.1)	94(1)	113(1)	136(1)	162(1)
	Lafayette, IN	76.1(0.1)	77.4(0.1)	78.8(0.1)	80.6(0.1)	48.4(0.1)	50.3(0.1)	52.7(0.1)	55.9(0.2)	56(1)	77(1)	100(1)	127(1)
	Springfield, MO	77.2(0.1)	78.4(0.1)	79.5(0.1)	81.4(0.1)	51(0.1)	52.7(0.1)	54.9(0.1)	57.9(0.1)	82(1)	102(1)	121(1)	147(1)
Upper Midwest	Appleton, WI	74.8(0.1)	75.9(0.1)	77.4(0.1)	79.3(0.1)	44.4(0.1)	46.3(0.1)	48.9(0.1)	52.5(0.1)	30(1)	47(1)	70(1)	98(1)
	Lansing, MI	75(0.1)	76.2(0.1)	77.7(0.1)	79.6(0.1)	45.3(0.1)	47.4(0.1)	50(0.1)	53.6(0.2)	30(1)	49(1)	74(1)	105(2)
	Madison, WI	75.1(0.1)	76.3(0.1)	77.8(0.1)	79.6(0.1)	44.1(0.1)	46.2(0.1)	48.8(0.1)	52.4(0.2)	31(1)	49(1)	73(1)	101(1)
	St. Cloud, MN	74.9(0.1)	76.1(0.1)	77.5(0.1)	79.5(0.1)	40.9(0.1)	43.2(0.1)	45.9(0.1)	49.9(0.2)	23(1)	39(1)	60(1)	89(2)
	Waterloo, IA	75.6(0.1)	76.8(0.1)	78.2(0.1)	80.1(0.1)	44.6(0.1)	46.7(0.1)	49.3(0.1)	52.8(0.2)	41(1)	60(1)	83(1)	109(1)
South	Jackson, MS	78.5(0.1)	79.5(0.1)	80.5(0.1)	82.2(0.1)	57.4(0.1)	58.9(0.1)	60.9(0.1)	63.8(0.1)	140(1)	157(1)	177(1)	202(1)
	Plainview, TX	77.5(0.1)	78.6(0.1)	79.7(0.1)	81.7(0.1)	50.1(0.1)	51.7(0.1)	53.9(0.1)	57.3(0.1)	79(1)	102(1)	122(1)	152(1)
	Stephenville, TX	78.6(0.1)	79.7(0.1)	80.6(0.1)	82.4(0.1)	55.6(0.1)	57.3(0.1)	59.5(0.1)	62.8(0.1)	124(1)	144(1)	163(1)	193(1)
	Wichita, KS	78.4(0.1)	79.8(0.1)	81.1(0.1)	83.2(0.1)	50.9(0.1)	52.7(0.1)	54.8(0.1)	58.1(0.1)	89(1)	108(1)	126(1)	150(1)
Northern Rockies	Dickinson, ND	75.1(0.1)	76.5(0.1)	78.1(0.1)	81(0.2)	38.1(0.1)	40.2(0.1)	42.8(0.1)	47(0.2)	12(1)	26(1)	46(1)	77(2)
	Grand Island, NE	76.9(0.1)	78.3(0.1)	79.9(0.1)	82(0.1)	45.9(0.1)	47.8(0.1)	50.2(0.1)	53.7(0.1)	54(1)	75(1)	96(1)	120(1)
	Great Falls, MT	74.4(0.1)	75.9(0.1)	77.4(0.1)	80.1(0.1)	41(0.1)	43.1(0.1)	45.8(0.1)	50.1(0.2)	6(0)	17(1)	38(1)	71(2)
	Watertown, SD	75.3(0.1)	76.6(0.1)	78(0.1)	80.2(0.1)	41.3(0.1)	43.4(0.1)	46(0.1)	49.9(0.2)	27(1)	44(1)	65(1)	92(1)

**Southwest**	**Phoenix, AZ**	81.3(0.1)	82.4(0.1)	83.3(0.1)	84.9(0.1)	59.3(0.1)	61.3(0.1)	63.7(0.1)	67.3(0.2)	143(1)	162(1)	184(1)	213(2)
	**Richfield, UT**	74.1(0.1)	75.2(0.1)	76.6(0.1)	79(0.1)	40.2(0.1)	42.3(0.1)	45.1(0.1)	49.4(0.2)	3(0)	12(1)	33(1)	69(2)
	**Roswell, NM**	77.8(0.1)	78.9(0.1)	80(0.1)	82(0.1)	50.5(0.1)	52.1(0.1)	54.5(0.1)	58(0.1)	85(1)	107(1)	127(1)	158(1)
	**Sterling, CO**	76.8(0.1)	78.2(0.1)	79.8(0.1)	82.2(0.1)	43(0.1)	44.9(0.1)	47.3(0.1)	51.2(0.2)	33(1)	57(1)	81(1)	109(1)
**Northwest**	**Baker City, OR**	74.7(0.1)	76.1(0.1)	77.5(0.1)	80(0.1)	41(0.1)	42.9(0.1)	45.3(0.1)	49.2(0.2)	3(0)	11(1)	28(1)	63(2)
	**Jerome, ID**	76.1(0.1)	77.5(0.1)	79(0.1)	81.5(0.1)	43.4(0.1)	45.4(0.1)	47.8(0.1)	51.6(0.1)	14(1)	33(1)	57(1)	91(1)
	**Seattle, WA**	73.3(0.1)	74.1(0.1)	75.1(0.1)	77.1(0.1)	50.1(0.1)	51.4(0.1)	52.9(0.1)	55.7(0.1)	3(0)	9(1)	22(1)	53(2)
	**Tillamook, OR**	72.4(0.1)	73.1(0.1)	74.1(0.1)	76.2(0.2)	49.4(0.1)	51.5(0.1)	53.7(0.2)	57.4(0.2)	1(0)	3(0)	16(2)	51(3)
**West**	**Elko, NV**	74.6(0.1)	76(0.1)	77.3(0.1)	79.7(0.1)	39.2(0.1)	41.3(0.1)	44.1(0.1)	48.2(0.2)	3(0)	13(1)	31(1)	66(2)
	**Sacramento, CA**	77.1(0.1)	78.3(0.1)	79.7(0.1)	81.6(0.1)	52.9(0.1)	54.3(0.1)	56.3(0.1)	59.5(0.2)	39(1)	63(1)	95(2)	135(2)
	**Visalia, CA**	78.2(0.1)	79.3(0.1)	80.5(0.1)	82.1(0.1)	54.5(0.1)	56.2(0.1)	58.3(0.1)	61.6(0.2)	79(1)	103(1)	130(1)	163(2)

*** Past: 1981 – 2000; Early: 2015 – 2034; Mid: 2045 – 2064; Late: 2081 – 2100**
