# Supplementary material for: Projected heat stress challenges and abatement opportunities for U.S. milk production
Source: PLoS One. 2019 Mar 28;14(3):e0214665. doi: 10.1371/journal.pone.0214665 (PMC6438606; doi:10.1371/journal.pone.0214665)
Supplement: S4 Table — (PDF) [file pone.0214665.s012.pdf]

S4 Table. Mean cost-benefit ratios of heat abatement implementation under Representative Concentration Pathway 8.5. Cost-benefit ratios ≤ 1 (shaded) show marginal breakeven or profitability.

Climatic region	Locations	Early-21st Century			Mid-21st Century			Late-21st Century		
		Mod*	Hig	Int	Mod	Hig	Int	Mod	Hig	Int
Northeast	Montpelier, VT	4.0	3.1	4.3	2.0	1.5	1.9	1.4	0.9	0.9
	Providence, RI	1.6	1.2	1.4	1.2	0.7	0.7	1.5	0.7	0.4
	State College, PA	1.8	1.4	1.8	1.2	0.8	1.0	1.1	0.6	0.5
	Syracuse, NY	2.0	1.5	1.8	1.3	0.9	1.0	1.2	0.7	0.5
Southeast	Athens, GA	0.7	0.5	0.5	0.7	0.4	0.4	1.0	0.5	0.3
	Avon Park, FL	0.5	0.3	0.3	0.6	0.3	0.3	1.1	0.4	0.2
	Gainesville, FL	0.6	0.4	0.5	0.6	0.4	0.4	0.9	0.4	0.3
	Lynchburg, VA	1.0	0.8	0.9	0.8	0.5	0.6	0.9	0.5	0.4
Ohio Valley	Akron, OH	1.8	1.4	1.7	1.2	0.8	0.9	1.0	0.6	0.5
	Franklin, TN	0.8	0.6	0.7	0.8	0.5	0.5	1.0	0.5	0.4
	Lafayette, IN	1.2	0.9	1.0	1.0	0.7	0.7	1.1	0.6	0.5
	Springfield, MO	0.9	0.6	0.6	0.8	0.5	0.5	1.1	0.5	0.3
Upper Midwest	Appleton, WI	2.0	1.5	1.9	1.4	1.0	1.1	1.2	0.7	0.6
	Lansing, MI	1.8	1.3	1.8	1.3	0.9	1.0	1.1	0.7	0.6
	Madison, WI	1.8	1.3	1.7	1.3	0.9	1.0	1.2	0.7	0.6
	St. Cloud, MN	2.2	1.6	1.9	1.5	1.0	1.1	1.2	0.7	0.6
	Waterloo, IA	1.5	1.1	1.3	1.2	0.8	0.8	1.1	0.6	0.5
South	Jackson, MS	0.7	0.4	0.5	0.7	0.4	0.4	1.1	0.5	0.3
	Plainview, TX	0.9	0.6	0.5	0.7	0.5	0.4	0.9	0.4	0.2
	Stephenville, TX	0.7	0.4	0.3	0.7	0.4	0.3	1.1	0.4	0.2
	Wichita, KS	0.9	0.5	0.4	1.0	0.5	0.3	1.9	0.6	0.2
Northern Rockies	Dickinson, ND	2.8	1.9	1.7	1.9	1.1	0.8	1.8	0.9	0.4
	Grand Island, NE	1.2	0.8	0.7	1.1	0.6	0.5	1.4	0.6	0.3
	Great Falls, MT	3.7	2.6	2.4	2.0	1.3	1.0	1.4	0.7	0.4
	Watertown, SD	1.9	1.4	1.5	1.4	0.9	0.9	1.2	0.7	0.5
Southwest	Phoenix, AZ	1.2	0.4	0.2	2.1	0.5	0.1	3.4	0.7	0.1
	Richfield, UT	3.6	2.5	2.6	2.2	1.4	1.2	1.5	0.8	0.5
	Roswell, NM	0.9	0.6	0.5	0.7	0.4	0.3	1.0	0.4	0.2
	Sterling, CO	1.5	1.0	0.8	1.2	0.7	0.5	1.4	0.6	0.3
Northwest	Baker City, OR	3.7	2.5	2.4	2.2	1.4	1.2	1.3	0.8	0.5
	Jerome, ID	2.1	1.4	1.1	1.4	0.9	0.6	1.1	0.6	0.3
	Seattle, WA	7.5	5.8	6.5	3.6	2.7	2.8	1.7	1.1	1.0
	Tillamook, OR	18.7	15.1	20.8	5.4	3.9	4.7	2.0	1.3	1.1
West	Elko, NV	3.4	2.3	2.2	2.1	1.3	1.1	1.3	0.7	0.5
	Sacramento, CA	1.3	0.8	0.6	1.0	0.6	0.4	0.8	0.4	0.2

Visalia, CA	0.9	0.6	0.4	0.8	0.5	0.3	0.8	0.4	0.2

* Min = minimal; Mod= moderate; Hig = high; Int = intense
