# Supplementary material for: Projected heat stress challenges and abatement opportunities for U.S. milk production
Source: PLoS One. 2019 Mar 28;14(3):e0214665. doi: 10.1371/journal.pone.0214665 (PMC6438606; doi:10.1371/journal.pone.0214665)
Supplement: S5 Table — (PDF) [file pone.0214665.s013.pdf]

S5 Table. Mean annual net profit (or loss) of heat abatement implementation by regions and time period, under Representative Concentration Pathway 8.5.

Statistic	Climatic region	Early-21st Century			Mid-21st Century			Late-21st Century		
		Mod*	Hig*	Int*	Mod	Hig	Int	Mod	Hig	Int
Mean	Northeast	-40.15	-30.02	-56.98	-21.48	9.02	3.58	-17.50	40.70	117.27
	Southeast	58.39	151.70	156.15	52.79	185.38	266.29	7.67	159.59	437.70
	Ohio Valley	-4.69	31.89	21.62	7.80	70.63	90.59	-3.95	87.70	206.99
	Upper Midwest	-35.81	-21.54	-47.45	-20.87	10.98	2.19	-13.05	43.18	87.52
	South	29.47	110.62	185.03	26.89	137.77	290.48	-11.60	125.22	487.26
	Northern Rockies	-37.82	-20.58	-23.58	-25.51	10.25	48.07	-25.28	38.78	193.82
	Southwest	-21.08	40.31	199.71	-17.93	66.36	332.07	-48.77	67.49	590.96
	Northwest	-55.15	-50.66	-68.45	-44.44	-29.50	-24.88	-25.69	16.45	100.10
	West	-20.63	15.86	62.48	-4.13	55.73	166.85	5.82	107.33	379.53
Number of locations	Northeast	4	4	4	4	4	4	4	4	4
	Southeast	4	4	4	4	4	4	4	4	4
	Ohio Valley	4	4	4	4	4	4	4	4	4
	Upper Midwest	5	5	5	5	5	5	5	5	5
	South	4	4	4	4	4	4	4	4	4
	Northern Rockies	4	4	4	4	4	4	4	4	4
	Southwest	4	4	4	4	4	4	4	4	4
	Northwest	4	4	4	4	4	4	4	4	4
	West	3	3	3	3	3	3	3	3	3
Standard error	Northeast	5.16	8.53	10.69	6.08	12.77	23.58	5.61	9.56	44.68
	Southeast	25.63	54.08	60.61	12.91	43.93	74.73	5.86	20.95	101.82
	Ohio Valley	12.55	22.31	29.78	9.25	21.71	34.62	2.29	11.64	41.48
	Upper Midwest	2.44	4.33	4.77	2.29	4.73	6.06	1.11	3.39	6.61
	South	11.97	22.92	32.02	9.08	17.90	33.13	12.12	17.77	43.66
	Northern Rockies	9.06	16.70	27.26	7.65	17.54	35.94	4.53	10.28	41.67
	Southwest	13.59	39.75	168.37	19.51	35.53	200.11	31.17	27.79	249.75
	Northwest	5.80	10.91	19.75	7.65	16.24	36.85	6.48	20.16	72.25
	West	17.05	33.31	67.73	19.02	40.86	94.69	13.06	39.13	127.27

* Min = minimal; Mod = moderate; Hig = high; Int = intense
