# Supplementary material for: Projected heat stress challenges and abatement opportunities for U.S. milk production
Source: PLoS One. 2019 Mar 28;14(3):e0214665. doi: 10.1371/journal.pone.0214665 (PMC6438606; doi:10.1371/journal.pone.0214665)
Supplement: S6 Table — Two similar letters in the same row following milk production losses show no significant difference (α = 0.05). Cost-benefit ratios ≤ 1 (shaded) show marginal breakeven or profitability. (PDF) [file pone.0214665.s014.pdf]

S6 Table. Late-20th Century milk production losses, net present value, and cost-benefit ratios under different abatements. Two similar letters in the same row following milk production losses show no significant difference (α=0.05). Cost-benefit ratios ≤ 1 (shaded) show marginal breakeven or profitability.

Climatic region	Location	Milk production loss (kg/cow/year)				Net present value ($/cow/year)			Cost-Benefit ratio		
		Min*	Mod	Hig	Int	Mod	Hig	Int	Mod	Hig	Int
Northeast	Montpelier, VT	43 a	14 b	6 c	7 c	-65	-69	-101	10.7	9.0	12.9
	Providence, RI	157 a	66 b	34 c	23 c	-55	-54	-84	3.6	2.9	3.7
	State College, PA	131 a	52 b	25 c	23 c	-57	-57	-89	4.1	3.3	4.6
	Syracuse, NY	127 a	54 b	27 c	21 c	-58	-58	-89	4.4	3.5	4.6
Southeast	Athens, GA	751 a	408 b	248 c	156 d	-11	18	6	1.1	0.8	1.0
	Avon Park, FL	1728 a	995 b	617 c	561 d	62	140	118	0.6	0.5	0.6
	Gainesville, FL	1162 a	617 b	364 c	348 c	27	76	46	0.8	0.6	0.8
	Lynchburg, VA	342 a	162 b	88 c	62 d	-41	-31	-58	2.0	1.5	1.9
Ohio Valley	Akron, OH	139 a	55 b	26 c	21 c	-56	-56	-87	3.9	3.1	4.2
	Franklin, TN	628 a	349 b	215 c	166 d	-22	2	-21	1.3	1.0	1.2
	Lafayette, IN	315 a	160 b	92 c	78 c	-44	-35	-64	2.2	1.7	2.2
	Springfield, MO	557 a	311 b	191 c	112 d	-27	-7	-22	1.5	1.1	1.2
Upper Midwest	Appleton, WI	134 a	58 b	30 c	24 c	-57	-57	-88	4.3	3.4	4.5
	Lansing, MI	151 a	68 b	36 c	35 c	-56	-55	-87	3.9	3.0	4.2
	Madison, WI	166 a	79 b	44 c	37 c	-55	-53	-84	3.7	2.9	3.8
	St. Cloud, MN	128 a	61 b	34 c	23 c	-58	-58	-88	4.7	3.7	4.6
	Waterloo, IA	228 a	114 b	65 c	54 c	-51	-46	-76	2.9	2.2	2.9
South	Jackson, MS	1152 a	688 b	434 c	344 d	12	62	50	0.9	0.6	0.7
	Plainview, TX	521 a	303 b	182 c	24 d	-33	-13	-9	1.7	1.2	1.1
	Stephenville, TX	1069 a	661 b	426 c	117 d	2	48	86	1.0	0.7	0.6
	Wichita, KS	808 a	529 b	352 c	107 d	-21	12	36	1.3	0.9	0.8
Northern Rockies	Dickinson, ND	110 a	62 b	37 c	6 d	-61	-61	-86	6.4	4.6	4.5
	Grand Island, NE	397 a	235 b	149 c	54 d	-42	-29	-39	2.1	1.5	1.5
	Great Falls, MT	59 a	28 b	14 c	0 d	-63	-66	-95	9.8	7.2	7.9
	Watertown, SD	161 a	83 b	48 c	25 d	-56	-54	-81	4.1	3.1	3.6
Southwest	Phoenix, AZ	2261 a	1938 b	1464 c	135 d	-21	80	355	1.3	0.6	0.3
	Richfield, UT	57 a	23 b	8 c	0 d	-62	-65	-95	8.9	6.8	8.2
	Roswell, NM	604 a	365 b	221 c	15 d	-30	-4	11	1.5	1.0	0.9
	Sterling, CO	274 a	164 b	100 c	12 d	-50	-42	-54	3.0	2.0	1.9
Northwest	Baker City, OR	67 a	33 b	16 c	1 d	-62	-64	-93	8.9	6.4	7.0
	Jerome, ID	163 a	92 b	50 c	1 d	-56	-52	-73	4.4	3.0	2.9
	Seattle, WA	24 a	9 b	4 c	0 d	-67	-71	-103	20.8	16.8	20.1
	Tillamook, OR	6 a	2 b	1 b	1 b	-69	-74	-106	75.4	64.4	91.2

West	Elko, NV	71 a	34 b	15 c	0 d	-62	-63	-92	8.2	5.9	6.6
	Sacramento, CA	404 a	260 b	166 c	15 d	-43	-28	-26	2.3	1.5	1.3
	Visalia, CA	695 a	455 b	295 c	33 d	-28	1	29	1.5	1.0	0.8

* Min = minimal; Mod = moderate; Hig = high; Int = intense
