# Supplementary material for: Projected heat stress challenges and abatement opportunities for U.S. milk production
Source: PLoS One. 2019 Mar 28;14(3):e0214665. doi: 10.1371/journal.pone.0214665 (PMC6438606; doi:10.1371/journal.pone.0214665)
Supplement: S1 File — (PDF) [file pone.0214665.s015.pdf]

Daily dew-point temperature and relative humidity projection

We calculated daily dew-point temperature using the method developed by Key and Sneering [1]. Under the assumptions that daily dew point temperature can be approximated by daily minimum temperature and that the contemporary difference between monthly minimum temperature and monthly dew point temperature is conserved in the future, we calculated the average monthly difference between mean monthly minimum temperature and mean monthly dew point temperature, using observed monthly data for 1950 to 2000 ((1) obtained from the Parameter-elevation Regressions on Independent Slopes Model (PRISM) [2]. The PRISM data were point data interpolated from observations using inverse-distance squared weighting [3,4]. We then projected daily dew point temperature as described in (2).

$$\theta o_{gm} = \frac{\sum_{y=1950}^{2000} \mathrm{Tmino}_{gmy} - \mathrm{Tdewo}_{gmy}}{N} \quad (1)$$

$$\mathrm{Tdewp}_{gdmy} = \mathrm{Tminp}_{gdmy} - \theta o_{gm} \quad (2)$$

Where θo is the average monthly difference between mean monthly minimum temperature and mean monthly dew point temperature (°C), Tmino and Tdewo are the monthly minimum and dew-point temperatures (°C) obtained from PRISM, Tminp and Tdewp are the projected daily minimum and dew-point temperatures, and the subscripts are day *d* within month *m* and year *y* for location *g*. To evaluate the accuracy of the daily dew-point temperature prediction, we compared the predictions to PRISM-reported values for the historical periods 2000-2010.

We projected daily mean relative humidity using the Clausius-Clapeyron approximation for saturation vapor pressure and the assumption of a fixed enthalpy of vaporization [5](3).

$$Rh_{gdmy} = 100\exp\left(-\frac{L(Tmean_{gdmy} - Tdew_{gdmy})}{R_w Tmean_{gdmy} Tdew_{gdmy}}\right) \quad (3)$$

Where Rh and Tmean are the mean relative humidity (%) and mean temperature (°C), R_w is the gas constant for water vapor (R_w= 461 J/K per kg) and L is the fixed enthalpy of vaporization (L=2.453*10^6 J/kg).

Reference

1. Key N, Sneeringer S. Potential effects of climate change on the productivity of U.S. dairies. Am J Agric Econ. 2014;96: 1136–1156.
2. PRISM Climate Group. Descriptions of PRISM spatial climate datasets for the conterminous United States. In: Oregon State University. 2017. Available from: http://prism.oregonstate.edu/documents/PRISM_terms_of_use.pdf.
3. Shepard, D. A two-dimensional interpolation function for irregularly-spaced data. In: Proceedings of the 1968 23rd ACM national conference. New York, NY: ACM Press; 1968. pp. 517–524.
4. Huang F, Liu D, Tan X, Wang J, Chen Y, He B. Explorations of the implementation of a parallel IDW interpolation algorithm in a Linux cluster-based parallel GIS. Comput Geosci.; 2011;37: 426–434.
5. Lawrence MG. The Relationship between relative humidity and the dew-point temperature in moist air: A simple conversion and applications. Bull Am Meteorol Soc. 2005;86: 225–233.
