# Supplementary material for: Projected heat stress challenges and abatement opportunities for U.S. milk production
Source: PLoS One. 2019 Mar 28;14(3):e0214665. doi: 10.1371/journal.pone.0214665 (PMC6438606; doi:10.1371/journal.pone.0214665)
Supplement: S2 File. E — (PDF) [file pone.0214665.s016.pdf]

Estimation of D_d (daily ratio of the number of hours during which Temperature Humidity Index – THI – is above threshold THI – THIthresh –).

St-Pierre et al. [1] proposed that THI follows a sine distribution throughout the day, where the daily minimum THI is reached at 4:00 and the daily maximum THI is reached at 16:00. We used a sine model approximation to estimate D_d (1), as suggested by St-Pierre et al.

$$\left.\begin{array}{l} D_d = 0 \; for \; THIthresh > THImax_d \\ D_d = 1 \; for \; THIthresh < THImin_d \\ \text{ELSE} \\ D_d = \dfrac{\pi - 2 * \arcsin\left(\dfrac{THIthresh - THImean_d}{THImax_d - THImean_d}\right)}{2\pi} \; for \; THIthresh > THImean_d \\ D_d = \dfrac{\pi + 2 * \arcsin\left(\dfrac{THImean_d - THIthresh}{THImax_d - THImean_d}\right)}{2\pi} \; for \; THIthresh < THImean_d \end{array}\right\} \quad (1)$$

Where $THImean_d$ is the mean of $THImax_d$ and $THImean_d$ on day d.

Reference

1. St-Pierre NR, Cobanov B, Schnitkey G. Economic losses from heat stress by US livestock industries. J Dairy Sci. 2003;86: E52–E77.
