# Supplementary material for: Projected heat stress challenges and abatement opportunities for U.S. milk production
Source: PLoS One. 2019 Mar 28;14(3):e0214665. doi: 10.1371/journal.pone.0214665 (PMC6438606; doi:10.1371/journal.pone.0214665)
Supplement: S3 File — (PDF) [file pone.0214665.s017.pdf]

Cost-benefit ratio analysis.

Marginal milk income present value.

We used the 1998-2000 market year average price of \$0.31 /kg of milk [1] for Late-20th Century period and the 2008-2014 market year average price of \$0.41 /kg of milk [1] for the 21st Century periods. Although milk prices vary geographically and temporally, our objective was to evaluate the influence of climate on the economic feasibility of heat abatement. Therefore, we applied a mean milk price of \$0.41 /kg of milk equally across the U.S. We compounded the marginal milk income value annually.

Heat Abatement Costs Present Value.

Moderate heat abatement investments above minimal abatement include the construction and maintenance cost differences between a naturally and mechanically ventilated dairy barn, and the installation, operation, and maintenance of fans. The cost of a naturally ventilated freestall barn can vary significantly around \$215/m^2 [2] due to the high variability of design, permitting, environmental improvements, and construction material prices. Likewise, these factors can largely influence the cost difference between a naturally and mechanically ventilated barn, independently of the cost of the fans. Therefore, we considered a mean cost difference of 25% and explored the sensitivity of cost-benefit ratio to variable cost differences, with the assumption that maintenance costs are similarly different. We modeled the costs of the ventilation system after Meyer et al. [3] and Ferreira et al. [4] and assumed the use of 90 cm – 368 m^3/min – 0.5 kw – 7-yr lifetime wall-in exhaust fans, which cost \$800 on average [2] and have a 10% residual value with a straight-line depreciation. Assuming one fan is used for five stalls, the air exchange rate per stall would be 74 m^3/min, which is larger than the minimum 28 m^3/min required for heat stress release [5], hence putting the capital cost at \$160/stall. We estimated the capital cost for fan installation at \$80/stall and conducted a sensitivity analysis on the installation cost. Fan maintenance (cleaning and repair) was estimated at \$30/fan per year [5].

High heat abatement adds water sprinkling to moderate heat abatement. Based on current market prices and recommendations from Smith and Harner [6] and Harner et al. [7], we determined that sprinklers cost an average of \$19.5/stall for purchase and installation and have a water consumption of 64 L/d per stall, with a 5-yr lifetime and 10% residual value (\$0.73/stall). We

assumed that sprinklers were annually serviced at a cost of $3/m of feedline, therefore $2.44/stall, assuming 123 stalls/100 m of feedline.

Intense heat abatement investments add evaporative cooling to moderate heat abatement. We considered the use of livestock-housing evaporative cooling systems with a capital cost estimated at $130/stall – 10-yr lifetime – null residual run by a 0.047 kw/stall pump. The installation costs of evaporative cooling system have not been previously documented, therefore we explored the sensitivity of the cost-benefit ratio to its variation. We assumed that heat abatement was operated for 24 h only on heat stress days (mean daily THI>70). Operating costs were modeled using a 2012-2016 U.S. mean electricity cost of $0.1042/kwh [8] and a mean water cost of $0.21/1000 L [4]. Information was not available for mean daily water use of the evaporative cooling system either, so we explored the production sensitivity to its variation. For maintenance, the evaporative cooling pads were considered replaced every three years at a cost of $51.25/stall.

Reference

1. USDA-NASS. Milk production report. 2017. Available from: http://future.aae.wisc.edu/data/monthly_values/by_area/3712?area=US.
2. Kammel DW. Building costs estimates - Dairy modernization. Univ. Wisconsin; 2015. Available from: http://fyi.uwex.edu/dairy/files/2015/11/Building-Cost-Estimates-Dairy-Modernization.pdf.
3. Meyer MJ, Smith JF, Harner JP, Shirley JE, Titgemeyer EC, Brouk MJ. Performance of lactating dairy cattle in three different cooling systems. Appl Eng Agric. 2002;18: 341–34.
4. Ferreira FC, Gennari RS, Dahl GE, De Vries A. Economic feasibility of cooling dry cows across the United States. J Dairy Sci. 2016;99: 9931–9941.
5. Gooch CA, Timmons MB, Karszes J. Economics of tunnel ventilation for freestall barns. 2000 ASAE Annual International. Milwaukee, WI: ASAE; 2000; Paper No. 004101.
6. Smith JF, Harner JP. Strategies to reduce the impact of heat and cold stress in dairy cattle facilities. In: Collier RJ, Collier JL, editors. Environmental physiology of livestock. Ames, IA: John Wiley & Sons, Inc; 2012. p. 277.
7. Harner JPI, Smith JF, Brook M, Murphy JP. Sprinkler systems for cooling dairy cows at a feed line. Manhattan, KS: Kansas State University, Agricultural Experiment Station and Cooperature Extension Service; 1999. MF-2401.
8. USEIA. Electricity data browser. In: Electricity data. Available from: https://www.eia.gov/electricity/data/browser/.
